# Geology of the InSight landing site on Mars

**DOI:** 10.1038/s41467-020-14679-1

**Published:** 2020-02-24

**Authors:** M. Golombek, N. H. Warner, J. A. Grant, E. Hauber, V. Ansan, C. M. Weitz, N. Williams, C. Charalambous, S. A. Wilson, A. DeMott, M. Kopp, H. Lethcoe-Wilson, L. Berger, R. Hausmann, E. Marteau, C. Vrettos, A. Trussell, W. Folkner, S. Le Maistre, N. Mueller, M. Grott, T. Spohn, S. Piqueux, E. Millour, F. Forget, I. Daubar, N. Murdoch, P. Lognonné, C. Perrin, S. Rodriguez, W. T. Pike, T. Parker, J. Maki, H. Abarca, R. Deen, J. Hall, P. Andres, N. Ruoff, F. Calef, S. Smrekar, M. M. Baker, M. Banks, A. Spiga, D. Banfield, J. Garvin, C. E. Newman, W. B. Banerdt

**Affiliations:** 1 Jet Propulsion Laboratory, California Institute of Technology, Pasadena, CA 91109 USA; 2 SUNY Geneseo, Department of Geological Sciences, 1 College Circle, Geneseo, NY 14454 USA; 3 Smithsonian Institution, National Air and Space Museum, 6th at Independence SW, Washington, DC 20013 USA; 4 DLR, Institute of Planetary Research, Rutherfordstr. 2, Berlin, 12489 Germany; 5 Laboratoire de Planétologie et Géodynamique, CNRS URM6112, Université de Nantes, Nantes, France; 6 Planetary Science Institute, 1700 E Fort Lowell, Suite 106, Tucson, AZ 85719 USA; 7 Department of Electrical and Electronic Engineering, Imperial College, South Kensington Campus, London, SW7 2AZ UK; 8 Division of Soil Mechanics and Foundation Engineering, Technical University of Kaiserslautern, Kaiserslautern, 67663 Germany; 9 Royal Observatory of Belgium, Avenue Circulaire 3, Brussels, 1180 Belgium; 10 Laboratoire de Météorologie Dynamique (LMD/IPSL), Sorbonne Université, Centre National de la Recherche Scientifique, École Normale Supérieure, École Polytechnique, Paris, France; 11 Institut Supérieur de l’Aéronautique et de l’Espace (ISAE-SUPAERO), Université de Toulouse, Toulouse, 31400 France; 12 Université de Paris, Institut de physique du globe de Paris, CNRS, F-75005 Paris, France; 13 Morton K. Blaustein Department of Earth and Planetary Sciences, Johns Hopkins University, 301 Olin Hall, 3400 N. Charles St, Baltimore, MD 21218 USA; 14 NASA Goddard Space Flight Center, 8800 Greenbelt Road, Greenbelt, MD 20771 USA; 15 Institut Universitaire de France, Paris, France; 16 420 Space Sciences, Cornell Center for Astrophysics and Planetary Science, Cornell University, Ithaca, NY 14853 USA; 17 Aeolis Research, 333N Dobson Road, Unit 5, Chandler, AZ 85224-4412 USA

**Keywords:** Planetary science, Geomorphology

## Abstract

The Interior Exploration using Seismic Investigations, Geodesy and Heat Transport (InSight) spacecraft landed successfully on Mars and imaged the surface to characterize the surficial geology. Here we report on the geology and subsurface structure of the landing site to aid in situ geophysical investigations. InSight landed in a degraded impact crater in Elysium Planitia on a smooth sandy, granule- and pebble-rich surface with few rocks. Superposed impact craters are common and eolian bedforms are sparse. During landing, pulsed retrorockets modified the surface to reveal a near surface stratigraphy of surficial dust, over thin unconsolidated sand, underlain by a variable thickness duricrust, with poorly sorted, unconsolidated sand with rocks beneath. Impact, eolian, and mass wasting processes have dominantly modified the surface. Surface observations are consistent with expectations made from remote sensing data prior to landing indicating a surface composed of an impact-fragmented regolith overlying basaltic lava flows.

## Abstract (web summary)

The InSight spacecraft landed on Mars on November 2018. Here, the authors characterize the surficial geology of the landing site and compare with observations and models derived from remote sensing data prior to landing and from ongoing in situ geophysical investigations of the subsurface.

## Introduction

The InSight spacecraft landed successfully in western Elysium Planitia on Mars on November 26, 2018^1^. Because the lander carries a payload focused primarily on exploring the interior of the planet, the regional setting and subsurface structure of the landing site provides important context for interpreting the scientific results of the mission. Furthermore, this landing represents only the 8th in situ evaluation of Martian geology (preceded by Viking Lander 1 and 2, Mars Pathfinder, Mars Exploration Rover Spirit and Opportunity, Phoenix, and Mars Science Laboratory) and the 1st along the planetary dichotomy in Elysium Planitia^2,3^. Significant uncertainty remains regarding the geologic history and origin (and age) of lowland plains material here^4^. The landing region was mapped in orbital images as the Early Hesperian Transition unit (eHt), which could be effusive volcanics or sedimentary^4^; either interpretation is important for the geologic history of the dichotomy and the northern plains.

**Fig. 1:**
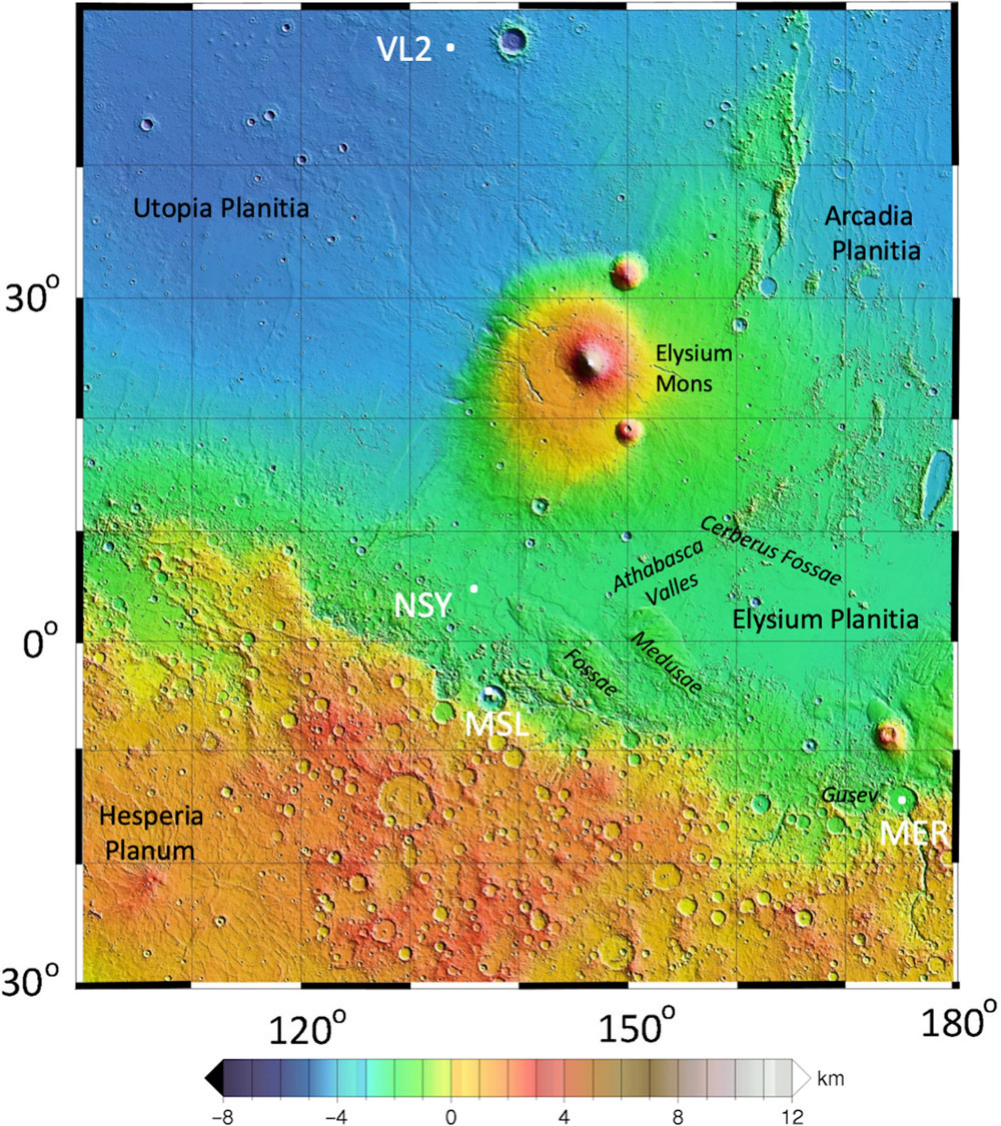
Topographic map of the region around the InSight landing site. The map shows InSight (NSY) and major physiographic features as well as the landing sites of the Viking Lander 2 (VL2), Mars Science Laboratory (MSL) Curiosity in Gale crater, and the Mars Exploration Rover (MER) Spirit in Gusev crater. InSight landed near the dichotomy boundary between the heavily cratered highlands to the south and the northern lowlands. Volcanic flows from Elysium Mons flowed to the south and very young lavas from Cerberus Fossae flowed down Athabasca Valles to 150 km to the east of the lander. The map is a portion of the MOLA shaded relief topographic map of Mars with elevations with respect to the geoid.

**Fig. 2:**
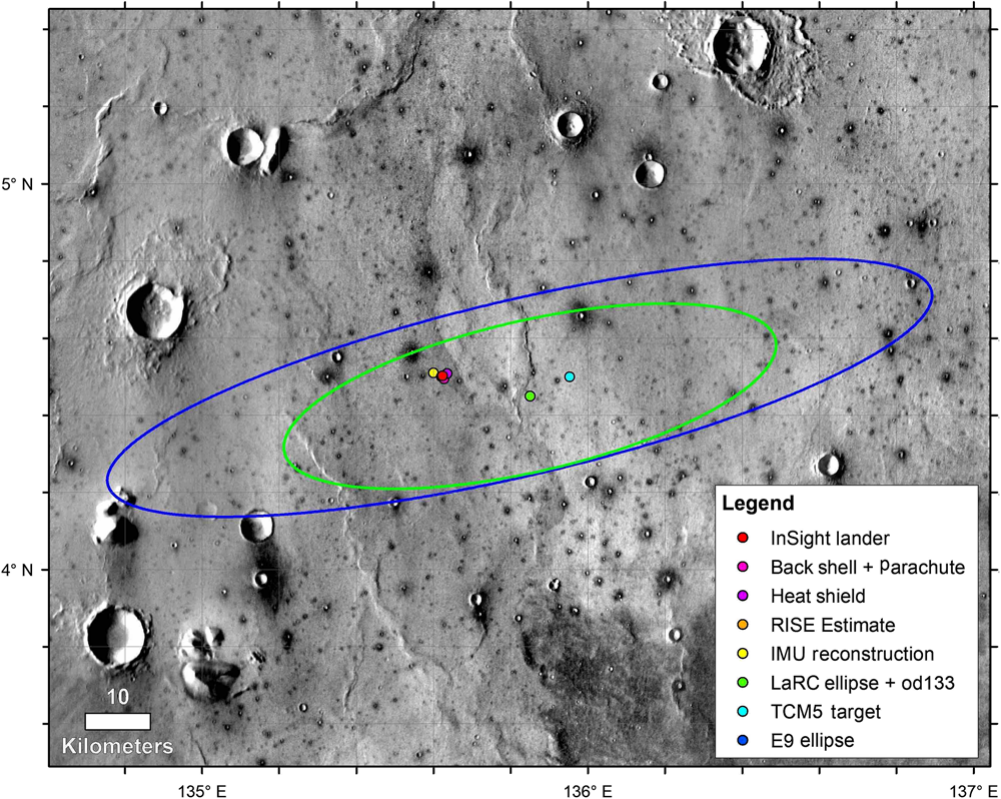
InSight landing ellipses and spacecraft locations. Image shows the landing ellipse (E9, dark blue, 130 km × 27 km), with trajectory correction maneuver 5 (TCM5) course adjusted target (green dot), the last orbit determination solution and ellipse (LaRC green, 77.4 km × 23.2 km), the extrapolated inertial measurement unit (IMU) surface location, the RISE estimate from Sol 1 (4.49751° ± 0.00471°N, 135.6178693° ± 0.000337°E, hidden behind the lander dot, red), and HiRISE-based location from December 6th, 2018. Background image mosaic is from the daytime Thermal Emission Imaging System (THEMIS) infrared global mosaic at 100 m/pixel. The dominant surface is smooth Early Amazonian-Hesperian plains deformed by north-trending wrinkle ridges (suggesting subsurface basalt flows) with large impact craters^2,3^. Craters larger than around 40 m but smaller than around 2 km are dark (indicating colder daytime temperatures with higher thermal inertia), rocky ejecta craters^18^. These craters excavate strong coherent rock (basalt) from depths of 4–200 m depth, with a fractured regolith on top and weaker sediments beneath^2,3,18^.

The landing site is near the dichotomy boundary^2,3^, which is interpreted as an area of intermediate crustal thickness between ancient Noachian heavily cratered highlands to the south and the younger northern lowlands^5^ (Fig. 1). The plains of western Elysium Planitia are located between highlands to the south and west, a ridge of Medusae Fossae Formation to the east and southeast, Hesperian and Amazonian lavas from Elysium Mons (the second largest volcanic complex on Mars) to the north, and very young lavas (the youngest ~2.5 Ma) from Cerberus Fossae, about 1500 km to the east, that flowed down Athabasca Valles^6,7^ to within 150 km of the lander (Fig. 1). Cerberus Fossae is among the youngest fault scarps on Mars with boulder trails attributed to paleomarsquakes^8^ and seismic events that were expected^9^ and have been observed by InSight^10^. Geologic mapping performed as part of the landing site selection process (prior to landing), indicates the plains beneath the lander (Fig. 2) formed from Early Amazonian-Hesperian lava flows that are about 200 m thick^2,3^ and are underlain by weaker phyllosilicate bearing sedimentary rocks of likely Noachian age^11^.

The InSight mission allows the direct comparison between surficial geology and in situ geophysical investigations on Mars. The scientific results from the geophysical payload are dependent on understanding the properties of the shallow subsurface beneath the lander that can be inferred from the local geomorphology and distribution of surface materials. The Seismic Experiment for Internal Structure (SEIS)^12^ seismometer measures accelerations that travel through the shallow subsurface, so the elastic and physical properties of these materials are important as inputs for models. Passive SEIS monitoring of atmospheric disturbances also yield information on subsurface properties^3,13^. In addition, the spacecraft carries a mole (part of the Heat Flow and Physical Properties Package, HP^3^), designed to percussively penetrate up to 5 m through unconsolidated material beneath the surface^14^ while SEIS records the hammering^13^, allowing the direct measurement of P-, and S-wave velocities and elastic properties^13,15^. Finally, the spacecraft also has a precision tracking system (Rotation and Interior Structure Experiment, RISE)^16^, which will determine the location of the lander in inertial space to about five times better than any previous lander on Mars.

Because of the HP^3^ mole, the landing site selection for InSight included specific requirements on the physical properties of the shallow subsurface (in addition to all of the usual engineering constraints of elevation, latitude, ellipse size, a radar reflective and load bearing surface, surface slopes, and rock abundance)^2^. As a result, the physical properties of the shallow subsurface were investigated using a wide variety of imaging and radar data during the landing site selection process^2,17,18^. These data indicated that the surface should be composed of dominantly sand (fine sand) with low rock abundance. This impact fragmented, unconsolidated regolith is about 3–18 m thick and overlies coarse breccia that grades into jointed basalt^2,3,18^.

In addition to the SEIS and HP^3^, the lander carries two color cameras, the lander mounted, Instrument Context Camera (ICC) and the arm-mounted, Instrument Deployment Camera (IDC)^19^. The IDC is attached to the forearm of a four degree of freedom Instrument Deployment Arm (IDA), used to deploy the instruments onto the surface, which also includes a scoop at the end^20^ that can interact with surface materials^3^. The cameras have acquired a large number of color surface images, including: stereo coverage at two resolutions (0.5 and 2 mm per elevation posting) of the instrument deployment workspace to select the locations to place the instruments, three complete stereo panoramas (morning, afternoon, and evening), and stereo images of the lander, its footpads, terrain under the lander, and the radiometer spots^21^ (Supplementary Tables 1 and 2, Supplementary Figs. 1–3). The HP^3^ includes a Radiometer (RAD)^14^ that measures the surface brightness temperatures in two fields of view facing north (opposite of the workspace). These measurements have been used to determine the thermal inertia of surface materials^22^, which can be related to soil grain size and/or cementation. InSight also measures wind speed and direction continuously, offering an opportunity to correlate surface bedforms, dust devils, and high winds with eolian changes imaged at the surface and to determine the threshold friction wind stress for grain motion on Mars^23^. Taken together, the instruments on InSight provide a lander-based, integrated view of the geology and physical properties of the surface and subsurface of Mars that can be compared and tested against observations and models derived from remote sensing data prior to landing^2,3,24^.

Here we report on the geology and shallow subsurface structure of the landing site based on observations from the lander and instruments and modifications created during landing. InSight landed in a degraded impact crater with a smooth sand-, granule- and pebble-rich surface. Slightly rockier and rougher terrain can be seen elsewhere. Craters in a variety of degradational states are common on the landscape and sparse eolian bedforms are visible at a distance sequestered adjacent to large, relatively fresh impact craters. During landing, pulsed retrorockets removed surficial fine-grained dust to create a darker surface, and scoured loose sand and granules away from the lander. Shallow pits beneath the lander and around the partially penetrated mole have steep slopes whose walls are composed of small rocks and pebbles cemented in a finer-grained matrix (duricrust). These observations indicate a near surface stratigraphy of surficial dust, over thin unconsolidated sand, underlain by a variable thickness duricrust, with poorly sorted, unconsolidated sand with rocks beneath. Thermal inertia measurements from the surface and orbit indicate surface materials are dominantly composed of fine sand. Properties of the surfaces and landforms indicate impact, eolian, and mass wasting processes have modified the surface. Observations by the lander are consistent with an impact-fragmented regolith overlying Hesperian-Early Amazonian basaltic lava flows inferred from remote sensing data prior to landing and ongoing geophysical investigations of the subsurface.

## Results

### Landing Location and Setting

**Fig. 3:**
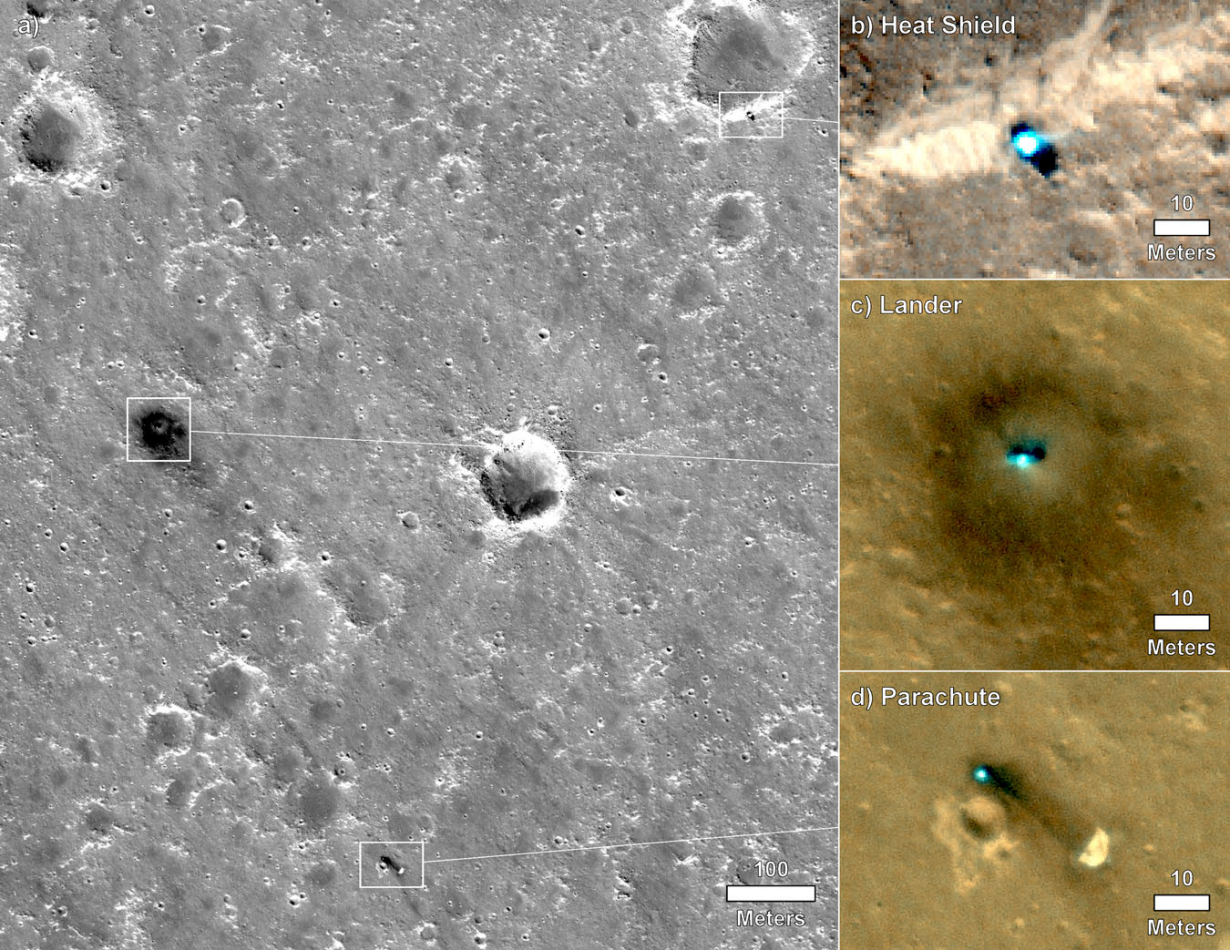
HiRISE image of InSight. **a** Image acquired on December 6, 2018 showing a regional view of the location of the InSight lander, parachute and backshell, and heatshield. Also shown are close ups of the heat shield (**b**) lander (**c**) and parachute and backshell (**d**) in color. Note the 20 m radius dark spot around the lander, with the slightly brighter interior. The gradational extension of the dark spot to the southeast is along the prevailing wind direction from the northwest estimated from orbit^3^ and measured by InSight early in the mission^41^. Note smaller dark spots associated with the backshell and heatshield, and the relatively fresh Sunrise crater 400 m to the east of the lander. Note circular impact craters in a wide variety of degradational states. Portion of HiRISE image ESP_057939_1845and ESP_058005_1845 at ~25 cm/pixel.

InSight landed near the center of the landing ellipse (130 km by 27 km)^2^ at 4.502°N, 135.623°E at an elevation of −2613.43 m (Figs. 2 and 3) in the Mars Orbiter Laser Altimeter, MOLA cartographic grid as imaged by the High-Resolution Imaging Science Experiment (HiRISE)^25^ (see Methods HiRISE and Doppler locations, Supplementary Figs. 4–7, Supplementary Tables 3 and 4). The distance to the RISE inertial location determined from X-band radio tracking from the first 34 sols of the mission is ~220 m to the west (Supplementary Figs. 4 and 7), which is a measure of the cartographic map tie uncertainty with inertial coordinates. This offset is similar to previous measurements on Mars^26,27^ and is important for landing spacecraft (which are tracked in inertial space) and improving the map tie uncertainty.

**Fig. 4:**
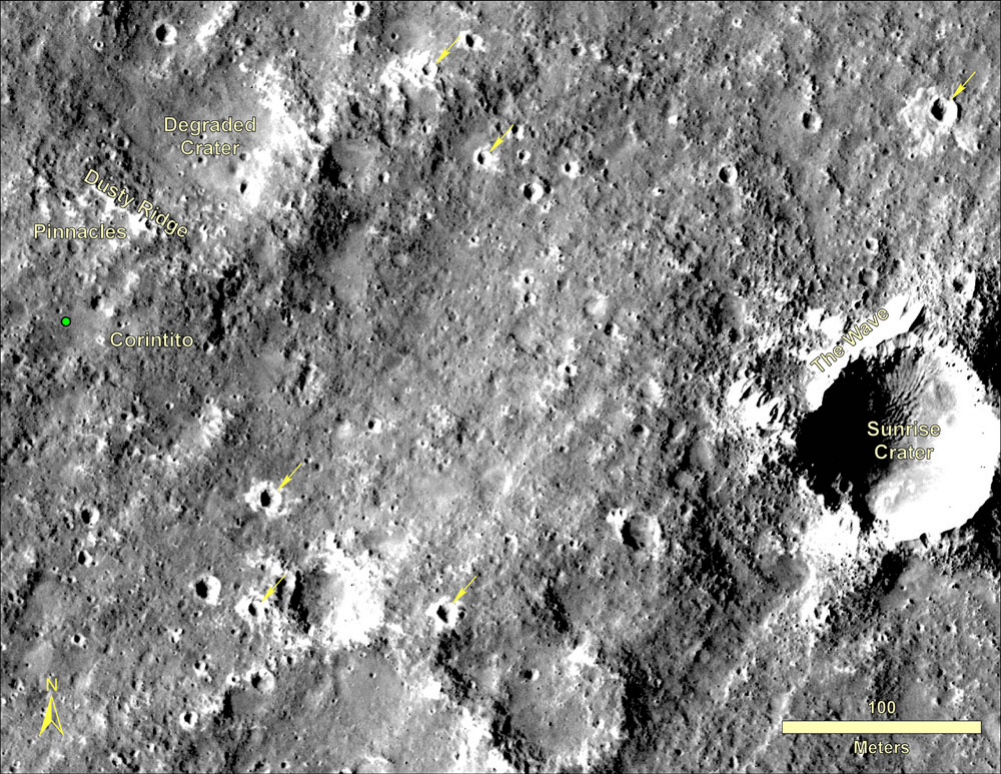
HiRISE image of region in view from the lander. Image shows the lander (green dot), and rocks, craters and bedforms observed in surface images. Note the relatively fresh, rocky ejecta crater Sunrise (~100 m diameter) about 400 m to the east and nearby bedforms observed from the lander (The Wave) that are typically near the rim or inside craters. Panoramas show terrain about 50 m to the north to the rim of a degraded crater (~100 m diameter) where bedforms (Dusty Ridge) and rocks (The Pinnacles) can be seen. Also note Corinto secondary craters (five yellow arrows and Corintito) with their characteristic bright ejecta. Portion of HiRISE image ESP_036761_1845.

**Fig. 5:**
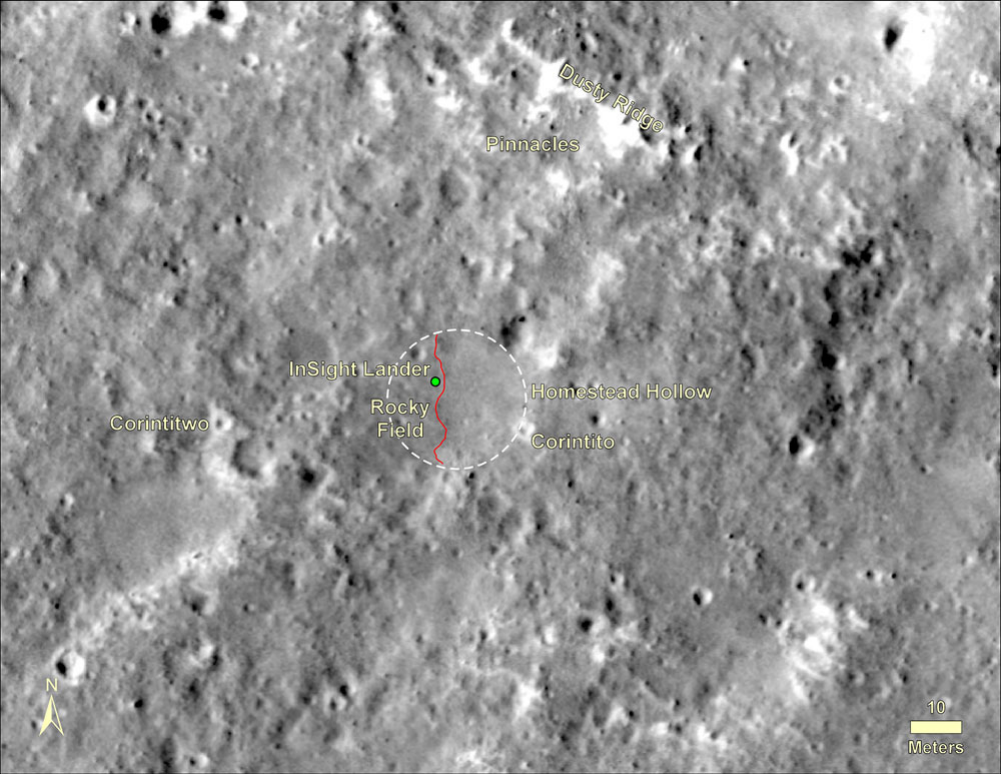
HiRISE image of Homestead hollow. Image shows the location of the InSight lander (green dot) in Homestead hollow (white dashed circle) and surface features identified from the ground. Note smooth terrain to the east of the lander and slightly rougher and rockier terrain (Rocky field) to the west (red line is the contact) and throughout much of the image. Bedforms (Dusty ridge) and three rocks (The Pinnacles) are about 50 m away to the north-northeast (see Fig. 4). Note two Corinto secondary craters that can be seen from the lander: Corintito (20 m to the southeast) and Corintitwo (40 m to the west). Portion of HiRISE image ESP_036761_1845.

**Fig. 6:**
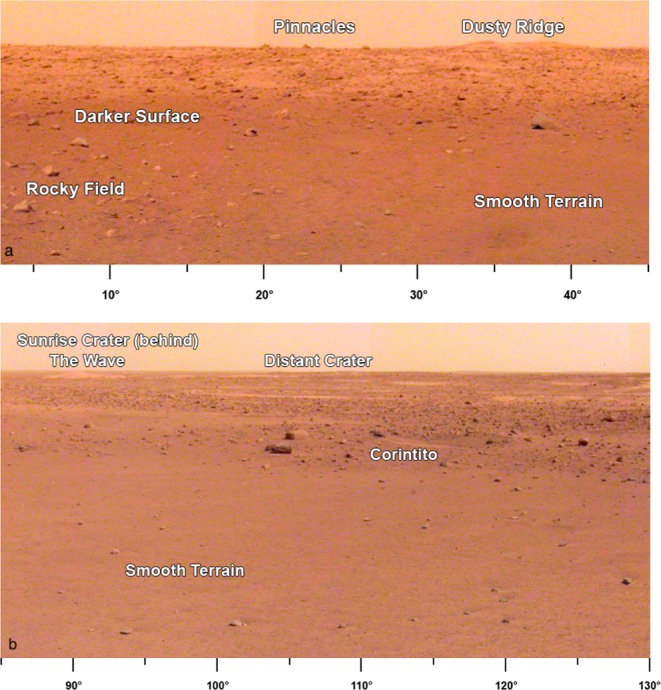
Portion of panoramas around the lander. **a** Panorama is the area to the north-northeast of the lander (azimuths below image). Note darker surface where dust has been removed within 20 m of the lander, rockier surface to the west (Rocky field), and smooth terrain to the east. Note The Pinnacles rocks and Dusty Ridge, which is an eolian bedforms on the southern edge of a degraded impact crater, located about 50 m north of the lander. **b** Portion of panorama to the east-southeast (azimuths below image) of the lander showing smooth terrain to the edge of Homestead hollow and rougher and rockier terrain beyond. Note fresh Corinto secondary crater (Corintito) on the edge of the hollow, circular soil filled depressions (hollows) in the distance, and eolian bedforms (The Wave) and Sunrise crater rim on the horizon about 400 m away. The rim of a larger (460 m diameter), relatively fresh crater (Distant crater) to the east-southeast is ~2.4 km away.

The lander is located on the western side of a quasi-circular depression (Supplementary Fig. 5), interpreted to be a degraded ~27 m diameter impact crater^28^, informally named Homestead hollow (Figs. 4–6), with a smooth, sandy, granule- and pebble-rich surface adjacent to slightly rockier and rougher terrain to the west. About ten, 1–10 m diameter impact craters can be seen in panoramic images within 20 m of the lander. Some of these craters have little relief and are filled with fine grained material. Farther afield, bright circular patches or hollows interpreted to be soil-filled, degraded craters are common (Fig. 6b). At least one fresh crater has the characteristic bright ejecta of Corinto secondary craters (impacted between 0.1–1 Ma and 2.5 Ma) that are ubiquitous across the landing ellipse^2^ (Figs. 5 and 6b). A slope to the north limits the horizon to about 50 m away (Supplementary Fig. 5); it is topped by three rocks (The Pinnacles), and eolian bedforms (Dusty ridge) near the southwest rim of a ~100 m diameter degraded impact crater (Figs. 3–5). To the east-southeast (Fig. 4), the horizon extends about 400 m to the rim of a relatively fresh, ~100 m diameter impact crater (Sunrise) with large eolian bedforms on its rim (The Wave). The rim of a larger (460 m diameter), relatively fresh crater can be seen on the east-southeast horizon ~2.4 km away (Distant Crater in Fig. 6b).

### Terrains

**Fig. 7:**
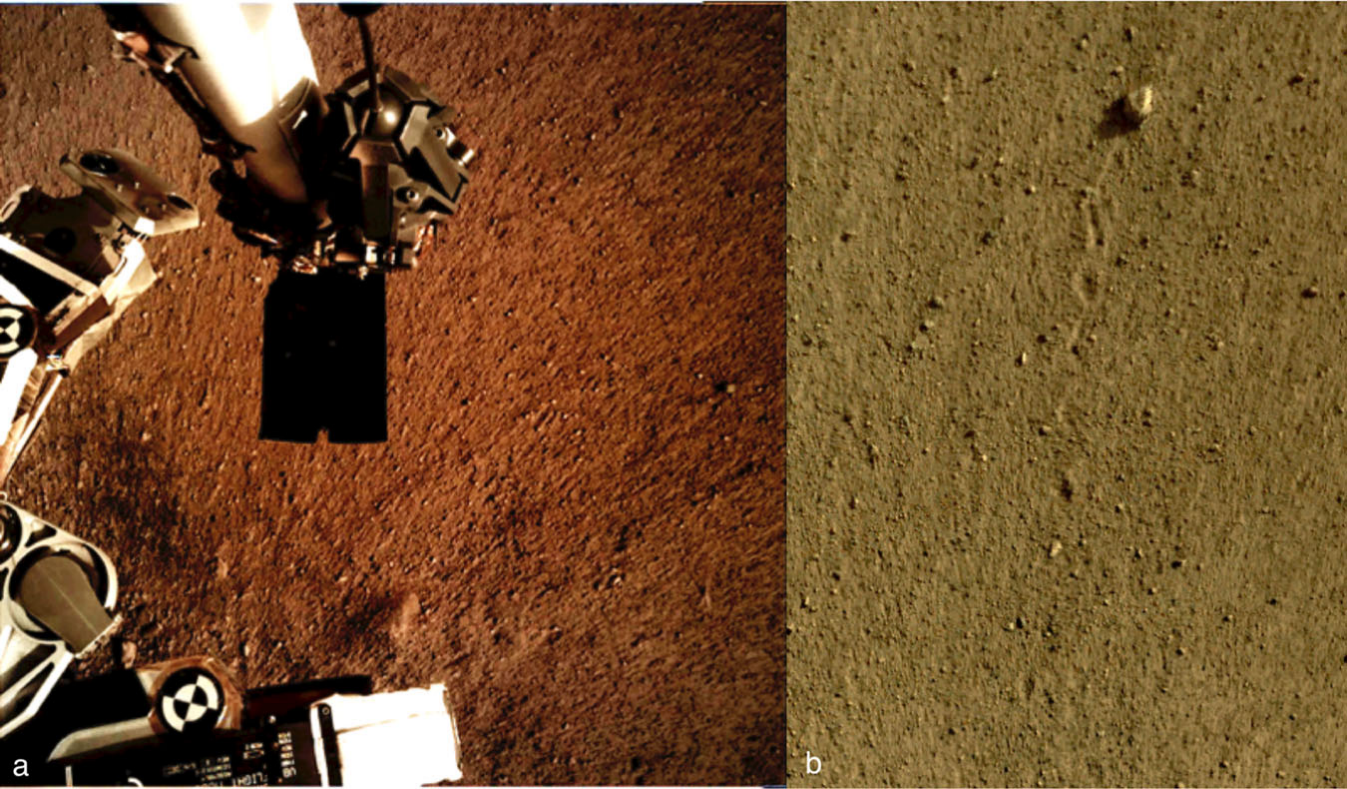
IDC images of the soil surface near the lander. **a** Image shows the radial striations in the soil. High resolution digital elevation models show millimeters of relief between the ridges and grooves^44^. Some elongate hills have pebbles at the lander facing end suggesting they protected the tails of material behind. The radial pattern and tails behind pebbles suggests dispersal of mostly unconsolidated sand away from the lander by the retrorockets. The lack of evidence for more significant scour around larger rocks suggests that only millimeters of sand has been removed around the lander (which would have minimal impact on clast and rock counts). The dark rectangle in the center of the image is the scoop at the end of the arm, which is 7.1 cm wide. The horseshoe shaped notch in the front blade of the scoop can be seen in the scoop indentation in Fig. 8b. **b** Image shows surface divots that record the displacement of the ~5 cm diameter pebble named Rolling Stones Rock. Approximately 10 divots show the pebble skipped and rolled about 1 m across the surface. The divots indicate the soils are fine grained and unconsolidated.

**Fig. 8:**
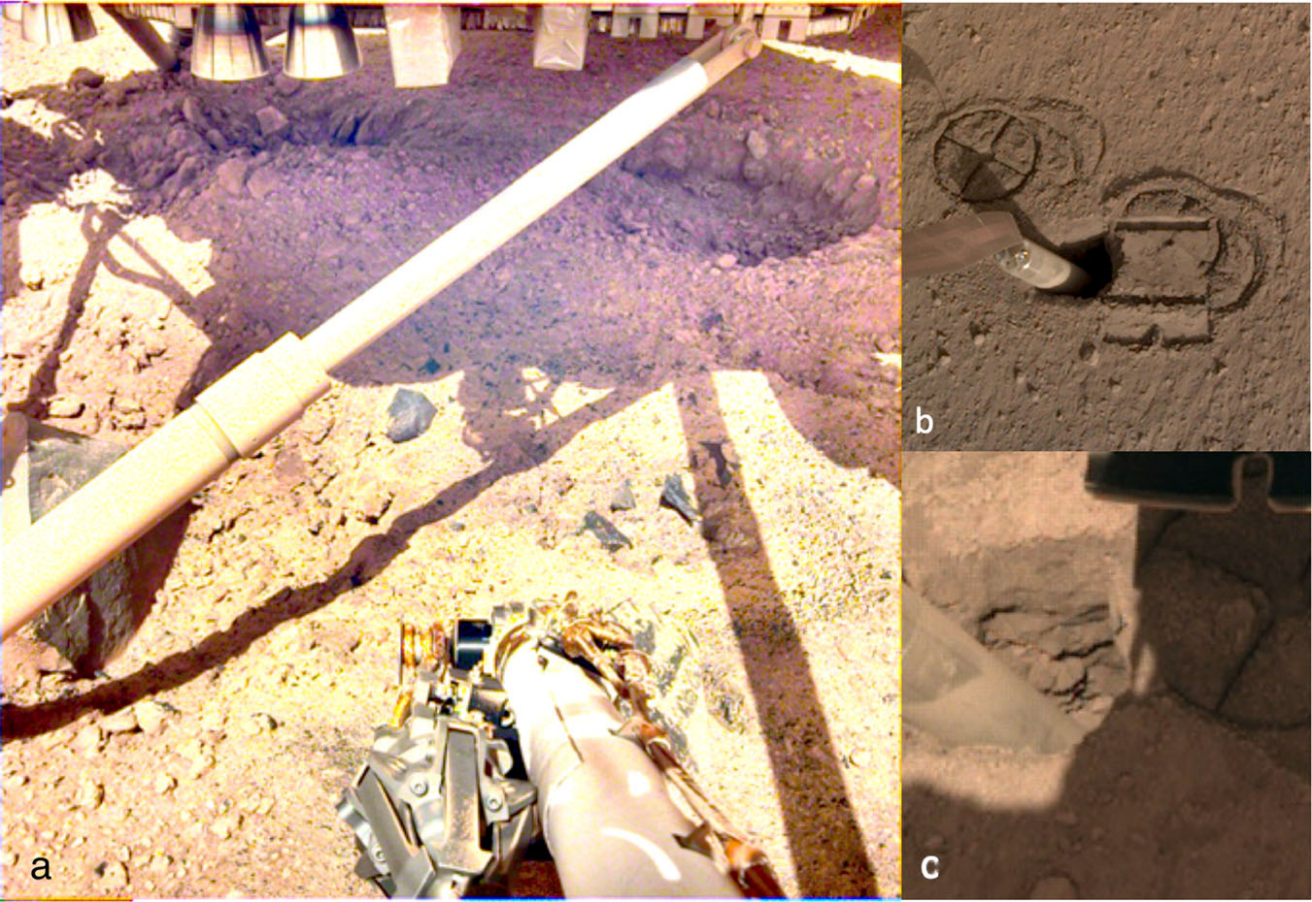
Images of shallow subsurface structure. **a** Image shows pits under the lander with spacecraft strut, retrorockets, excavated pits (~10 cm deep, ~50 cm across), dark gray, very fine-grained rocks (basalt) and duricrust. Note steep pit walls of soil and clasts in a finer-grained matrix, indicating cemented duricrust and clods and fragments of the duricrust that litter the pits and surface. Pulsed retrorockets on the Phoenix lander eroded 5–18 cm of material beneath the lander^42^. A contrast stretch has been applied to this image to accentuate details in the shadowed areas. **b** Image of mole hole and surface after interactions with the HP^3^ SSA feet and scoop. Circular cross patterns are imprints of the HP^3^ SSA feet in the soil. Smooth, reflective rectangular surface is where the flat base of the scoop (7.1 cm wide) was pressed against the soil, causing a ~5 mm indentation. Note the horseshoe shaped outline of the front blade of the scoop imprint (Fig. 7a). Horizontal troughs near the top and bottom of the scoop imprint are where the front blade of the scoop penetrated into the soil. **c**) Image of hole created by the HP^3^ mole showing resistant layers in the wall of the pit. These layers have steep edges and overhangs indicating cohesion in the soil. Small rocks appear cemented in a fine-grained matrix, similar to the pits beneath the lander. Mole is angled 2.7 cm diameter cylinder (~15°), to the left.

The surface of Homestead hollow is smooth with few rocks and the resolvable particle size distribution from the lander is dominated by granules and pebbles^29^ (Fig. 7). Cobble and pebble shape and form are equant to sub-equant and angular to sub-angular^29^. Clast counts of the granules and pebbles range in diameters from 1 to 7 mm^29^, but along with rocks only cover a small fraction of the surface indicating that most of the smooth terrain is composed of sand-sized particles (as indicated by thermal inertia measurements discussed later). Many of the larger rocks closest to the lander have a dark gray color and appear very fine grained (Fig. 8), consistent with aphanitic, dark mafic rocks (basalts). This is consistent with mapping results prior to landing and provides an important constraint for evaluating the geomorphology and origin of other northern lowland locations, including previously visited landing sites^2,3^. Other rocks appear lighter as if covered by dust and/or weathering rinds. At least one rock appears fluted (ventifacted), suggesting eolian abrasion. No obvious eolian bedforms (e.g., dunes or ripples) have been identified closer to the lander than those adjacent to the craters 50 and 400 m away.

To the west of the lander, the surface is slightly rougher and rockier than the smooth hollow (Fig. 5) and this rougher terrain extends into the distance at most azimuths away from the lander. Rocky Field, west of the lander (Fig. 5), has more, larger rocks and a rougher surface (Fig. 6a). Although rock abundance is about 2 times higher than on the smooth terrain (see Methods Rocks), the soil is granule- and pebble-rich and otherwise appears similar to the smooth terrain.

Rock abundance is generally low and in agreement with expectations prior to landing^2^. Rock counts were performed in the instrument deployment workspace in the smooth terrain closest to the lander to the south, further south of the lander (5 m) in the rocky terrain with the largest rocks close to the lander, in rocky terrain to the northwest of the lander, and in the near and far RAD spots to the north-northwest of the lander (see Methods Rocks). Cumulative fractional area versus diameter size-frequency distributions for rocks 6–20 cm diameter are similar to exponential rock size-frequency models for 1–4% rock abundance that have been used to describe rock populations for evaluating landing sites on Mars^30,31^. At diameters smaller than 4 cm, the rock distributions are steeper than the exponential models and cover 1–3% of the area. The largest rock in the far RAD spot is <5 cm and ~2% of the surface is covered by rocks >3 cm, which are too small and cover too little area to have any appreciable effect on the derived thermal inertia (see Methods Rocks). The rock distributions and abundance in the smooth and rocky terrain, are most similar to the ~2% measured at the Phoenix landing site^32^ and the 4% measured at the Spirit landing site^33^. These three landing sites are also the most granule- to pebble-rich landing sites on Mars and have steep distributions for rocks smaller than 5 cm, which may be due to deflation where finer particles have been removed by the wind, similar to the Spirit landing site^33^.

### Origin and evolution of homestead hollow

Homestead hollow has a similar morphology and soil characteristics to the degraded, sediment-filled impact craters on the Gusev cratered lava plains^33,34^. A morphometric analysis (e.g., depth, rim height, slope) of 2261 craters (>20 m diameter) in a 20 km^2^ region surrounding InSight, including 1316 Homestead hollow-like quasi-circular depressions, confirm an impact origin^28^. The data indicate that the hollows are part of a morphologic continuum^35^ that is caused by progressive crater rim destruction and infilling. The size frequency distribution of all craters in the dataset with diameters between 20 and 100 m (including the hollows) follows a power-law slope that is consistent with a −2 crater equilibrium slope^36,37^. From this distribution, the crater retention age of Homestead hollow is ~400–500 Myr^28,38^.

The morphology of the hollow records degradation by eolian and impact processes and lesser mass wasting that is similar to what is observed in Gusev hollows^33,34,39^. Formation of small simple craters such as Homestead hollow results in a landform surrounded by ejected fragments of varying size^34^. Characterizing the relative abundance or deficiency of coarse fragments standing in relief reflects net removal or deposition of fines, respectively. As a result, the relative abundance of perched, embedded, and buried rocks can be used as an indicator of where finer material has been removed or deposited. An impact creates a landform out of equilibrium with local geomorphic thresholds and leads to early stripping of fines from the exposed margin and their subsequent deposition within the crater. The result is more exposed or perched rocks on the rim (~70% relative to a combined 30% buried and embedded rocks) and fewer large, but predominantly buried and embedded rocks inside the hollow (58% relative to 42% perched rocks)^40^ (Supplementary Note 1, Supplementary Fig. 8). Early degradation likely included gravity-driven slope (mass wasting) processes as rocks were shed from the rim to the floor, though burial by fines masks their occurrence at the surface. Subsequent impacts (e.g., Corintito and others) directly modify the hollow via excavation, emplacement of ejecta, and enable short pulses of additional infilling as ejecta is stripped by the wind. Eolian degradation is limited by very slow weathering and breakdown of resistant basaltic rim blocks (supported by the occurrence of ventifacts) that continues at a greatly diminished average rate. The origin of Homestead hollow as a degraded impact crater suggests that the crater is dominantly filled with eolian sand that is ~3 m thick (based on an initial depth/diameter ratio of 0.15)^28,35^. This interpretation is supported by the greater number of seismically detected atmospheric convective vortices^13^, with low pressure centers that pull the ground up during passage^41^, to the east. This suggests the shallow subsurface to the east is weaker (composed of unconsolidated sand) than the terrain outside the hollow to the west (Supplementary Note 2, Supplementary Fig. 9).

### Near-surface stratigraphy

During landing, the pulsed retrorockets disturbed the surface^42^ under and around the lander, providing views into subsurface materials and their physical properties. HiRISE images acquired roughly a week after landing show a large dark spot centered on the lander^43^ (Fig. 3). The dark spot extends ~20 m away from the lander to the north and is distinctly darker than the surrounding surface (~35% lower relative albedo), but the transition is more gradational to the south. The inner 5 m of the dark spot in HiRISE images is slightly brighter than the rest of the spot. In the workspace near the lander, the surface appears striated and scoured, with multi-millimeter relief ridges and troughs that extend radially away from the lander^44^ (Fig. 7). Some pebbles and protrusions have tails extending away from the lander. One pebble skipped and rolled about 1 m across the surface creating divots and elongated depressions (Fig. 7b). These observations are consistent with the pulsed descent rocket exhaust removing surficial fine-grained dust to create the dark spot and scouring unconsolidated sand, granules and intermixed dust to produce the slightly brighter inner part^43^.

Retrorockets excavated three pits up to ~10 cm deep beneath the lander offering a unique view below the surface not available at other landing sites (Fig. 8a). In one pit, the exposed subsurface material is poorly sorted with pebbles and cobbles. Two pits have steep slopes (greater than the angle of repose; up to 60°–70°) composed of small rocks and pebbles cemented in a finer-grained matrix (duricrust)^45^. Slope stability analysis indicates that minimum cohesions of 5–24 Pa are sufficient to maintain the pit slopes (Supplementary Note 3, Supplementary Figs. 10–12). Smaller clods and pieces of this material are scattered within the pits and adjacent to the pits. One footpad appears partially buried by the material excavated from the pit. Two footpads show evidence for slight sliding into place, creating a depression on one side and a bulge in the direction of travel.

Interactions between the HP^3^ Surface Support Assembly (SSA)^14^, mole, and the scoop with the soil also provide a unique opportunity to evaluate the mechanical properties of martian soil in the context of geologic observations. At the time of this writing, the 2.7 cm diameter mole partially penetrated into the ground and is tilted within a steep-sided open pit ~5 cm wide and ~5 cm deep (Fig. 8b). Circular cross imprints of the underside of the HP^3^ feet and the very smooth and reflective imprint of the flat base of the scoop on the surface suggests the topmost surface materials are composed of sand, granules and intermixed fine dust and is similar to imprints of rover wheel tracks^46^. The open pit and lack of significant piles of excess soil around the hole suggests the underlying soils are low density and/or porous and were compressed by the mole hammering. The imprint of the scoop at the surface is around 0.5 mm deep and the lack of slumping into the pit from the load imparted indicates the cohesion of materials adjacent to the pit must be at least 1–1.9 kPa for soils with reasonable bulk densities and angles of internal friction (Supplementary Note 3). Laboratory tests using the mole showed open pits formed in simulants^47^ with cohesions of 2.5–12.5 kPa. The north wall of the pit shows relatively horizontal, resistant layers with vertical edges and overhangs (Fig. 8c). Some of the layers have pebbles that appear cemented in a finer-grained matrix. These steep, resistant layers are similar to the duricrust observed in the pits beneath the lander and the clods of material scattered during landing. The layers of crust and duricust could be cemented by salts deposited by thin films of water via interactions of atmospheric water vapor and soils as suggested by chemical measurements by Viking and Mars Exploration Rover spacecraft^48–50^.

**Fig. 9:**
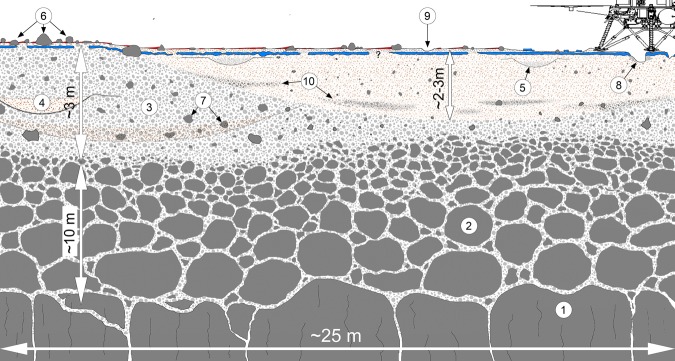
Interpretive cross section of the shallow subsurface beneath the InSight lander. Most of the surficial bright, reddish dust (red, shown behind some rocks) has been dispersed around the lander (above 8). Rockier areas beyond ~20 m have more surface dust (6). The dust, which settled out of the atmosphere, is likely microns thick. About 1 cm of unconsolidated sand indicated by the radial surface striations and surface divots (9) underlies the dust. Observed in the pits beneath the lander (8) and in the mole hole is a duricrust of cemented sand, pebbles and rocks that is 5–10 cm thick (shown in blue), but could vary in thickness. Beneath the duricrust are overlapping craters (4, 5), rocks (7), and lens of ejecta from other craters (10). The relatively fine-grained impact generated regolith (3) is around 3 m thick beneath the lander and likely grades with depth into coarse, blocky ejecta (2) that overlies fractured basalt flows (1). Observations from the lander described in the text support the top 10 cm of the cross section. The bottom 13 m of the cross section are derived from estimates of the thickness of the relatively fine-grained regolith from rocky and non-rocky ejecta craters^2,3,18^ and the original depth of the Homestead hollow crater. Note the varying vertical scale.

These observations suggest a near-surface stratigraphy (Fig. 9) of surficial dust that is microns thick (removed within 20 m), over ~1 cm of thin unconsolidated sand, underlain by a variable thickness (cm) duricrust, with poorly sorted, unconsolidated sand and rocks beneath (perhaps in quasi-continuous horizons related to materials ejected from nearby impacts). Orbital^2^ and lander radiometer^22^ measurements of thermal inertia (160–230 J m^−2^ K^−1^ s^−1/2^) indicate a surface dominated by fine sand size particles (~150 micron for an average thermal inertia of ~200 J m^−2^ K^−1^ s^−1/2^) (Supplementary Note 4, Supplementary Fig. 13). The rock size and abundance within the RAD field of view is too low to influence the thermal inertia and the lack of pronounced seasonal variations in the orbital derived thermophysical properties suggests an absence of steep thermophysical contrast in the top few tens of centimeters^2^. Thermal modeling also limits the volume of cement to a fraction of a percent. All of these observations (the duricrust, unconsolidated sand, and low rock abundance) are consistent with the relatively low seismic velocities observed as well as the elastic properties indicated from the seismic data during mole hammering^13^. The soils observed at the landing site are generally similar to soils at other landing sites on Mars^51–53^, and their origin via impact and eolian processes is likely similar to the Spirit landing site^54^.

## Discussion

The terrains and surface features in view of the lander include craters in various stages of degradation and dusty eolian bedforms. Based upon the origin and modification of Homestead hollow and adjacent impact craters, slow mass wasting and eolian processes punctuated by impacts are the dominant processes modifying the surface. No outcrop or bedrock has been observed. The low rock abundance and thermal inertia measurements indicate a surface dominated by sand sized particles that can be produced by impact and eolian activity^54^. The only eolian bedforms that have been observed from the surface (ripples) are adjacent to large impact craters. This is consistent with observations in high-resolution orbital images that show almost all bedforms (dunes and ripples) are sequestered inside or near the rims of relatively fresh impact craters and their orientations are consistent with modeled and measured winds^3,23,41^. These bedforms are bright and the surface is dusty, indicating little recent eolian activity. These observations suggest that the surface is old and has largely reached aerodynamic equilibrium with surface winds.

The terrains and surface materials observed by the lander are generally as predicted from remote sensing data prior to landing^24^. Orbital investigations indicated a surface composed of >3 m thick impact-fragmented regolith^18^ overlying Hesperian to Early Amazonian basaltic lava flows that would be similar to the Spirit landing site^2,3,18^. The terrains observed and the materials present at the site formed dominantly by impact, mass wasting, and eolian processes that highlights the importance of non-aqueous processes shaping the martian surface today. These processes created an impact-generated regolith composed mostly of sand-sized particles with variable pebbles, cobbles and boulders associated with a sequence of degraded impact craters that overlie basalt flows (Fig. 9). This subsurface stratigraphy is consistent with initial seismic investigation of Mars’ interior^13^ and will be further tested and refined by future InSight observations and measurements.

## Methods

### HiRISE Location of the InSight Lander

InSight landed near the center of the E9 landing ellipse^2^ (130 km by 27 km) (Fig. 2). Using the initial radio tracking location, HiRISE on the Mars Reconnaissance Orbiter spacecraft acquired images of the lander, heatshield and backshell/parachute on December 6 and 10, 2018 (Fig. 3). In carefully hierarchically georeferenced images and digital elevation models^2^, from high resolution (HiRISE, 0.25 m/pixel) to lower resolution (Context Camera, CTX, ~6 m/pixel, and High-Resolution Stereo Camera, HRSC, 12.5 m/pixel), all referenced to the Mars Orbiter Laser Altimeter, MOLA cartographic grid (463 m/pixel) and geoid^55^, the lander is located at 4.502°N, 135.623°E at an elevation of −2613.43 m (Figs. 2 and 3) in the northwest-central portion of the landing ellipse in western Elysium Planitia^25^. The distance to the RISE^16^ inertial location determined from X-band radio tracking from the first 34 sols of the mission is ~220 m west (Supplementary Fig. 4), which is a measure of the cartographic map tie uncertainty with inertial coordinates in this part of Mars and is similar to previous measurements of this offset^26,27^.

The lander is 12 km west-northwest from the last Orbit Determination (OD) (post Trajectory Correction Maneuver, TCM-6) and 1.38 km from the surface location indicated by the Inertial Measurement Unit (IMU) determined a few days after landing. Fig. 2 shows the E9 landing ellipse (blue)^2^ as well as the last OD solution (od133) and the appropriate ellipse to go with it (LaRC, green). The TCM-5 target is where the first HiRISE and CTX images were targeted (neither showed the lander). The December 6 image was targeted to the RISE location determined after tracking on sol 1^25^. Supplementary Fig. 4 shows a closer up view of the IMU location, and the RISE location determined after tracking on sol 1 and after 30 sols, and the location of the lander.

The lander, backshell/parachute and heatshield were localized on the carefully georeferenced HiRISE image^25^. The distance to the RISE location from first 30 sols of tracking is ~220 m west (Supplementary Fig. 4). The lander is 13.78 km from od133 solution (4.502384°N, 135.623447°E, Northing = 266877.460 m, Easting = 8039038.792 m, Elevation = −2613.426 m), but well within the landing ellipse (Fig. 2). The heatshield is located 0.762 km downtrack (northeast) from the lander, at an azimuth of 62.3° (4.508346°E, 135.634845°N, Northing = 267231.038 m, Easting = 8039715.141 m, Elevation = −2617.504 m). This position is visible from the lander, but the heatshield has not been identified. The backshell/parachute is located 0.553 km to the southeast at an azimuth of 152.3° (4.49413°N, 135.627781°E, Northing = 266388.697 m, Easting = 8039296.003 m, Elevation = −2614.012 m). The elevations are from a HiRISE stereo derived digital elevation model (InSightE17_C), hierarchically georeferenced to coarser digital elevation models and the MOLA grid, with an elevation uncertainty of ~0.2 m. Supplementary Fig. 5 shows a portion of this topographic map with the lander in Homestead hollow outlined, which has relief of 0.2–0.5 m.

### Estimation of InSight landed position from Earth-based Doppler

InSight was tracked using RISE after landing by the NASA Deep Space Network (DSN) for about 1 h each Martian day (sol). The measured Doppler shift is proportional to the rate of change of distance *ρ* between the tracking station and the lander. An approximate description of the Doppler shift is given by:^56^

1 $$\frac{{\partial \rho }}{{\partial t}} = \frac{{\partial \rho _{EM}}}{{\partial t}} + \frac{{\partial \rho _{DSN}}}{{\partial t}} - \frac{\partial }{{\partial t}}\,\, \left[ {R_z\sin \delta _E + R_s{\mathrm{cos}}\delta _E{\mathrm{cos}}\left( {{\it{\upphi }} + \lambda - {\it{\upalpha }}_E} \right)} \right]$$

where *ρ*_*EM*_ is the distance from the center of Earth to the center of Mars, *ρ*_*DSN*_ is the fraction of distance from the DSN tracking station to the center of Earth parallel to the Earth-Mars direction, *R*_*Z*_ is the distance of the lander from the martian equatorial plane, the spin radius *R*_*S*_ is the distance from the lander to the martian spin axis, *ϕ* is the angle of rotation of Mars about its spin axis, λ is the longitude of the lander, and *α*_*E*_ and *δ*_*E*_ are the right ascension and declination of Earth as viewed from Mars, where declination is the angle from the martian mean equatorial plane and right ascension is the angle in the martian equatorial plane measured from where the Sun crosses above the plane (martian vernal equinox).

Given knowledge of the orientation of the martian spin axis and rotation about the spin axis from previous lander and orbiter missions, the Doppler data can be used to estimate spin radius and longitude. The third cylindrical coordinate *R*_*z*_, and hence latitude, can be derived from matching *R*_*s*_ and λ to the Martian shape (topography) determined by MOLA^57^. The uncertainty in the estimated spin radius and longitude due to data noise and possible calibration errors is much less than the uncertainty due to the Mars rotation model. The major uncertainty in the Mars rotation model is the choice of reference longitude. The IAU Working Group on Cartographic Coordinates and Rotational Elements defines the reference longitude as the center of the crater Airy−0^58^. Unfortunately, the center of the crater is difficult to define exactly and most surface features from images have positions determined by ties to the MOLA topographic map that was derived with a previous determination of the center of Airy−0^55^.

For this paper, we have used a simplified expression for the rotation of Mars that can be easily seen to be close to the definition of longitude used in the MOLA data reduction, for the purpose of obtaining position estimates with Doppler data similar to those from images referred to MOLA. The model, given in Supplementary Table 3, matches the most recent estimate of the Mars spin axis and rotation rate variation within 3 × 10^−6^ ° over the time span from 1970 to 2030, but fixes longitude to be similar to that used by MOLA though adopting the same mean value (ignoring periodic terms) of the rotation angle *W* about the spin axis at the epoch J2000.0.

Supplementary Fig. 6 shows the difference in the location of the Mars prime meridian rotated to inertial space between the model used for MOLA data reduction and the model in Supplementary Table 3. The linear trend is due to improved estimation of the Mars rotation rate, and the periodic signature is due to seasonal variations in rotation rate from condensation/sublimation of CO_2_ at the poles. The prime meridian offset at epoch J2000.0 occurs due to the improvement in estimation of the Mars pole direction. This offset could be removed at J2000.0, or at the mean MOLA epoch, by adjusting the constant in the expression for *W* in Supplementary Table 3.

With this model, the position of the InSight estimate with Doppler data from only sol 1 and from the first 34 sols through December 31, 2018 are given in Supplementary Table 4 The uncertainties account for the noise in the Doppler data, uncertainty in the Mars rotation rate corresponding to displacement uncertainty of 3 cm/year in the longitude direction, uncertainty in the alignment of the MOLA topography to this rotation model in order to determine *R*_*z*_ from the observed spin radius and longitude, and uncertainty in determination of *R*_*z*_ arising from the discretization of the topography model with 128 points per degree^55^.

Supplementary Fig. 7 shows position estimates from the first sol of Doppler data after landing using different Mars rotation models. Only the first sol of data is used because error in the Mars rotation rate in the IAU 2009 model is not suitable for use with more than one sol of data. The longitude estimated using the IAU 2015 model is significantly offset from the estimate given in Supplementary Table 4 based on the rotation model in Supplementary Table 3, because of the different estimates of longitude of the crater Airy-0 used in the 2015 model. This offset in longitude results in a difference in estimated *R*_*z*_ coordinate, and hence latitude, derived from the MOLA topography. The positions estimated using the IAU 2009 model is close to the current estimate by design of the rotation model in Supplementary Table 3. The location derived from imaging in the cartographic grid from MOLA shown in Supplementary Fig. 7 is consistent with the position derived from Doppler data, but is offset in longitude by a significant amount.

### Rock abundance and rock size-frequency distributions

**Fig. 10:**
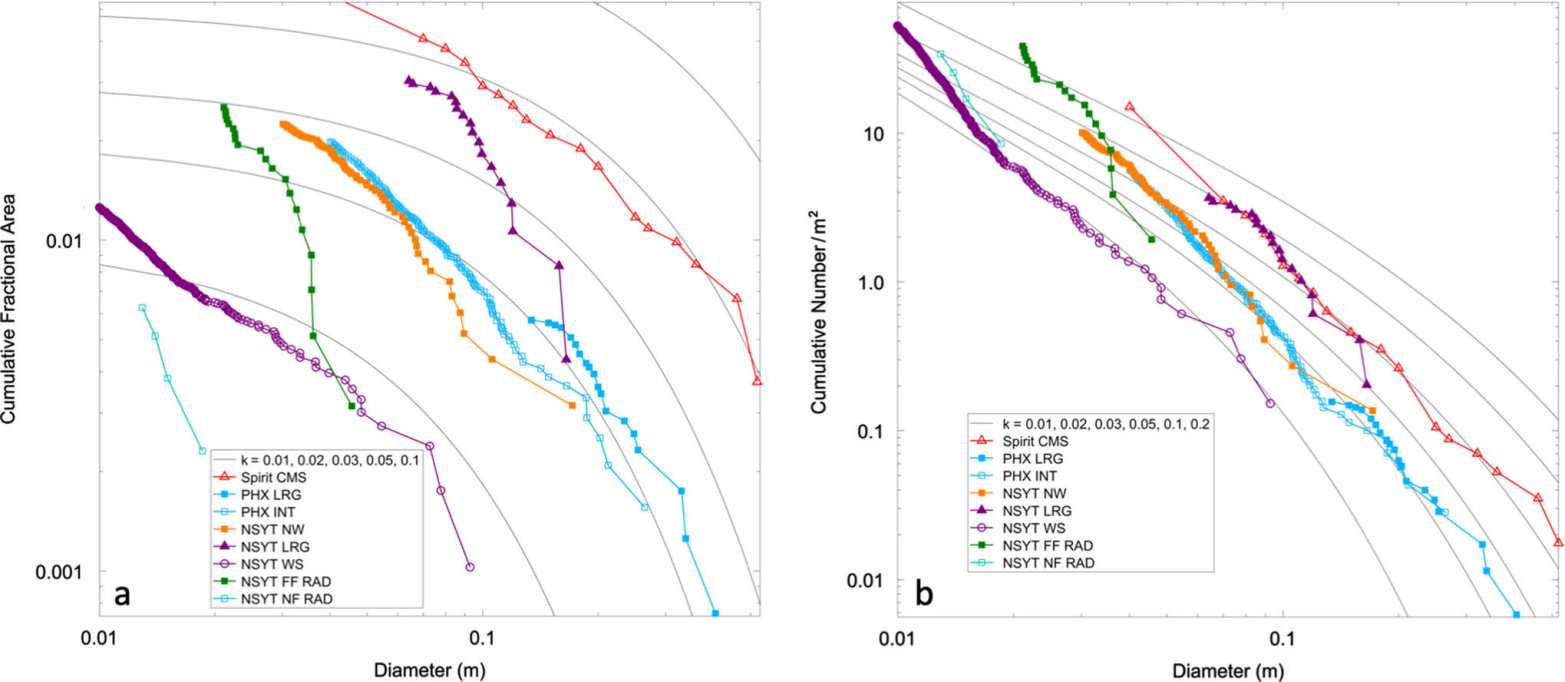
Rock size-frequency distributions. **a** Cumulative fractional area and **b** cumulative number per m^2^ versus diameter of rocks near the InSight lander as well as those measured at the Spirit (Spirit CMS for Columbia Memorial Station) and Phoenix (PHX) landing sites. Also shown are exponential model size-frequency distributions for rock abundances (k) of 1%, 2%, 3%, 5% and 10%^30^. Note curves in **b** are not exponentials and approach a straight line at small diameter (note that fractional area is dependent on the diameter squared, whereas cumulative number is not), but are matches to the exponential models based on cumulative fractional area in (a). Surface rock counts are: near and far RAD spots (NSYT NFF RAD and NSYT FF RAD, respectively), the workspace (NSYT WS), the area to the northwest (NSYT NW), and the area to the south with the largest rocks (NSYT LRG). Spirit CMS from Golombek et al.^33^, Phoenix intermediate area (PHX INT) from Heet et al.^68^ and Phoenix largest rocks (PHX LRG) from Golombek et al.^32^. Measurement uncertainty as stated earlier is between 1–4 mm, which would have no appreciable effect on the plots.

Rock counts were performed in the instrument deployment workspace (the smooth plains next to the lander to the south), farther to the south of the lander (5 m) in the rocky terrain with the largest rocks close to the lander, in rocky terrain to the northwest of the lander, and in the near and far RAD spots to the north-northwest of the lander (Fig. 10). These areas are representative of the rock abundance of the site, had stereo coverage to aid in measurement of rock size, or are areas observed by the HP^3^ RAD, which is important for interpreting the radiometer measurements and derived thermal inertia (Supplementary Note 4). The counts of the RAD measurement spots, concentrated on the larger rocks, as only rocks larger than 3 cm diameter begin to affect the thermal inertia^59^. These counts are of small areas and so do not characterize distributions over larger areas that are more representative of the actual rock distribution. They are provided for interpretation of the RAD measurements and derived thermal inertia only.

Surface rock abundance is typically measured using rock size-frequency distributions in which rock diameter of all rocks larger than a given size are measured over an area. The size-frequency distributions are typically plotted as the cumulative fractional area and/or cumulative number of rocks (normalized by area) versus rock diameter in log-log plots (Fig. 10).

Rock edges are digitized as polygonal outlines in orthorectified and oblique images using ArcMap. A convex hull is then calculated for each digitized rock for which there is topography derived from stereoscopic coverage^21^, providing a minimum axis and maximum axis for each rock in map space for orthorectified images (measured in meters) or pixel space for oblique images (measured as pixels). For orthoimages, such as the workspace mosaic, these axes represent the horizontal length and width of rocks. For oblique images, further processing is necessary to calculate the two oblique axes in meters. To convert oblique measurements from pixels to meters, two axes may be calculated as the product of the distance from the camera (in meters), the camera’s angular resolution of 0.8 milliradians/pixel, and the number of pixels for each axis calculated from convex hulls. Distance is calculated as the square root of the sum of the squares of the differences in (x,y,z) coordinates between each rock’s center as extracted from a stereo correlation map and the location of the camera derived from IDA telemetry and lander orientation. Minimum and maximum axes are averaged to yield the mean diameter for each rock. The cumulative fractional area (CFA) and cumulative number of the rocks are integrated over the count area as functions of diameter.

As an additional exercise, we also used MATLAB to extract all (x,y,z) points within the digitized polygon for each rock where there is sufficient stereo coverage and calculated a minimum bounding ellipsoid to those 3D points to yield 3 orthogonal axes (approximate length, width, and height) for each rock. Maximum and minimum axes calculated via this method bound but are typically similar to the maximum and minimum axes calculated using the 2D hulls, except for a small subset of high aspect ratio rocks with long axes pointed away from the camera.

The size-frequency distributions measured from landers and in HiRISE images (~0.3 cm/pixel) from orbit have been successfully fit to exponential rock size-frequency models that have been used to describe rock populations for landing spacecraft^2,30–32,59^. These models were derived from cumulative fractional area versus diameter plots that are curved on a log-log plot with exponentially fewer rocks with increasing diameter (Fig. 10a) and are generally similar to Rosin Rammler and Weibull distributions that have also been used previously to describe rock populations^59–64^. More recently, Charalambous^65^ has shown that repeated fragmentation events, each of which is scale invariant (fractal) or a power law^66^, results in a particle size-frequency distribution described by a negative binomial that resembles the exponential models. Rock counts in nearly complete HiRISE coverage of the InSight landing site, were fit by a negative binomial and predicted by the observed cratering^2^ and resulted in simulated surface and subsurface rock distributions that are consistent with observations at the surface^67^. Finally, a composite size-frequency distribution of particles (rocks to dust) can be explained by fragmentation due to impact for particles above 0.2–0.5 mm, with eolian activity responsible for the reduction below this size; together they can produce the global surface layer of mostly sand size particles on Mars^54^.

Rock counts were performed in five areas with varying rock density that have stereo digital elevation models produced early in the mission and include: the instrument deployment workspace in the smooth terrain closest to the lander to the south, further south of the lander (5 m) in the rocky terrain with the largest rocks close to the lander, in rocky terrain to the northwest of the lander, and in the near and far RAD spots to the north-northwest of the lander

The workspace of the arm, immediately south of the lander, is a crescent shaped area that the instruments can be deployed in. The area consists of smooth plains, interpreted as the infilled surface of the degraded Homestead hollow crater. The NYST WS count includes 692 rocks ranging from 1 cm to 10 cm that were measured over an area of 6.6 m^2^, which is the deployment workspace of the SEIS instrument. These rocks are located to the south of the lander in the 2 mm/pixel IDC orthomosaic (D_LRGBI0012_CPG010060ORRASB_F1MMWKSM2.VIC) from a digital elevation model created for instrument deployment projected in lander site frame. The measurement uncertainty is within a couple of pixels (2–4 mm).

The area to the northwest of the lander is composed of slightly rockier terrain. The NSYT NW count included 107 rocks ranging in size from 0.17 to 0.03 m in diameter, measured over an area of 7.36 m^2^ about 6.5 m to the northwest of the lander. The measurement uncertainty is estimated to be within 2–4 mm. Measurements were determined using stereo data from the pair D010L0014_597775998EDR_F0103_0100M2.VIC and D010R0014_597776396EDR_F0103_0100M2.VIC.

The area to the south of the lander (beyond the workspace) had the largest rocks close enough to the lander for high-quality digital elevation models to be created. The NSYT LRG count included 18 rocks ranging from 0.17 to 0.064 m in diameter, measured over an area of 4.92 m^2^. The measurement uncertainty is estimated to be within 2–4 mm. Measurements were determined using stereo data from the pair D015L0014_597778320EDR_F0103_0100M2.VIC and D015R0014_597778681EDR_F0103_0100M3.VIC.

The far RAD measurement spot is located about 4.5 m to the north-northwest of the lander. The NSYT FF RAD count included 41 rocks ranging from 0.05 to 0.005 m in diameter over an area of 1.08 m^2^. The measurement uncertainty is estimated to be within 2–4 mm. Measurements were determined using stereo data from the pair D010R0014_597776396EDR_F0103_0100M2.VIC and D010L0014_597775998EDR_F0103_0100M2.VIC.

The near RAD measurement spot is located about 0.5 m from the north-northwest edge of lander. The NSYT NF RAD count included 4 rocks ranging from 0.02 to 0.007 m in diameter over an area of 0.12 m^2^. The measurement uncertainty is estimated to be within 1–2 mm. Measurements were determined using stereo data from the pair D001L0018_598131526EDR_F0606_0010M2.VIC and D001R0018_598131636EDR_F0606_0010M2.VIC.

In the cumulative fractional area versus diameter plot (Fig. 10a), rocks in the workspace are smaller than 10 cm diameter and fall just below the 1% model to about 2 cm diameter, where the distribution become steeper and just exceeds 1% rock abundance at 1 cm diameter. Rocks to the northwest fall just below the 2% model for diameters of 20–7 cm; for smaller diameters the distribution rises to ~3% rock abundance at 2 cm diameter, and are similar to the rock size-frequency distribution at the Phoenix landing site. The largest rocks toward the south with diameters of >10 cm fall between the 2 and 3% model curves. At smaller diameters the rock distribution is similar to the Spirit landing site (~4%). The near and far RAD spots have no rocks larger than 2 cm and 5 cm, respectively. The near RAD spot has no rocks large enough to affect the thermal inertia (~3 cm) and the far RAD spot has about 2% of the surface covered by rocks >3 cm and so have no appreciable effect on the thermal inertia^59^. Taken together, the smooth terrain near the lander has a rock abundance of 1–2% and the rockier terrain has a rock abundance of 2–4%.

In the cumulative number per m^2^ versus diameter plot (Fig. 10b), rocks in the workspace are parallel to the 1% rock abundance model for diameters 2–10 cm, but rise sharply to 50 rocks/m^2^ at 1 cm, which is near the 10% rock abundance model. Rocks to the northwest of the lander are parallel to the 2% model for diameters larger than 7 cm and rise sharply at smaller diameters to the 10% rock abundance model at 3 cm diameter. This distribution is similar to that measured at the Phoenix landing site. The distribution of rocks to the south of the lander (the largest rocks close to the lander) fall between the 3% and ~5% models for diameters >10 cm and rise at smaller diameters to the 10% rock abundance model at 6 cm diameter. This distribution is similar to the Spirit landing site. All of the counts are pebble rich with the distributions rising more steeply than the model curves at smaller diameters.

## Supplementary information

Supplementary Information

## Data Availability

All data from NASA spacecraft are available in the NASA Planetary Data System archive. All InSight data discussed in this paper are in the Geosciences node at: https://pds-geosciences.wustl.edu/missions/insight/index.htm. All HiRISE and THEMIS data are in the Cartography and Imaging Node at: https://pds-imaging.jpl.nasa.gov/. The datasets generated during and/or analyzed during the current study are available from the corresponding author on reasonable request.

## References

[1] Banerdt, W. B. et al. Early results from the InSight mission: surface environment and global seismic activity. *Nat. Geosci*. 10.1038/s41561-020-0544-y (2020).

[2] Golombek M (2017). Selection of the InSight landing site. Space Sci. Rev..

[3] Golombek M (2018). Geology and physical properties investigations by the InSight Lander. Space Sci. Rev..

[4] Tanaka, K. L., et al. Geologic Map of Mars, 1:20,000,000, USGS Scientific Investigations Map 3292 (2014).

[5] Smrekar SE (2018). Pre-mission InSights on the interior of Mars. Space Sci. Rev..

[6] Burr DM (2002). Repeated aqueous flooding from the Cerberus Fossae: Evidence for very recently extant, deep groundwater on Mars. Icarus.

[7] Vaucher J (2009). The volcanic history of central Elysium Planitia: Implications for martian magmatism. Icarus.

[8] Brown JR, Roberts GP (2019). Possible evidence for variation in magnitude for marsquakes from fallen boulder populations, Grjota Valles, Mars. J. Geophys. Res.: Planets.

[9] Taylor J, Teanby NA, Wookey J (2013). Estimates of seismic activity in the Cerberus Fossae region of Mars. J. Geophys. Res. Planets.

[10] Giardini, D. et al. The seismicity of Mars. *Nat. Geosci.*10.1038/s41561-020-0539-8 (2020).

[11] Pan, L. et al. Crust stratigraphy and heterogeneities of the first kilometers at the dichotomy boundary in western Elysium Planitia and implications for InSight lander. *Icarus* **338**, 113511, 10.1016/j.icarus.2019.113511 (2020).

[12] Lognonné P (2019). SEIS: Insight’s seismic experiment for internal structure of Mars. Space Sci. Rev..

[13] Lognonné, P. et al. Initial results from SEIS with a focus on shallow Mars structure. *Nat. Geosci.*10.1038/s41561-020-0536-y (2020).

[14] Spohn T (2018). The heat flow and physical properties package (HP^3^) for the InSight mission. Space Sci. Rev..

[15] Kedar S (2017). Analysis of regolith properties using seismic signals generated by InSight’s HP^3^ penetrator. Space Sci. Rev..

[16] Folkner WM (2018). The rotation and interior structure experiment on the InSight mission to Mars. Space Sci. Rev..

[17] Putzig NE (2017). Radar-derived properties of the InSight landing site in western Elysium Planitia on Mars. Space Sci. Rev..

[18] Warner NH (2017). Near surface stratigraphy and regolith production in southwestern Elysium Planitia, Mars: implications for Hesperian-Amazonian terrains and the InSight lander mission. Space Sci. Rev..

[19] Maki JN (2018). The color cameras on the InSight lander. Space Sci. Rev..

[20] Trebi-Ollennu A (2018). InSight Mars lander robotics instrument deployment system. Space Sci. Rev..

[21] Abarca H (2019). Image data processing for the InSight landet operations and science. Space Sci. Rev..

[22] Mueller, N. T. et al. The HP3 radiometer on InSight. Ninth International Conference on Mars, Pasadena, California, July 22–25, 2019, Abstract #6194 (2019).

[23] Spiga A (2018). Atmospheric science with InSight. Space Sci. Rev..

[24] Golombek, M. et al. Initial assessment of InSight landing site predictions. 50th Lunar and Planetary Science, Abstract #1696 (2019).

[25] Parker, T. J. et al. Localization of the InSight lander. 50th Lunar and Planetary Science, Abstract #1948 (2019).

[26] Arvidson RE (2004). Localization and physical properties experiments conducted by Spirit at Gusev crater. Science.

[27] Arvidson RE (2004). Localization and physical properties experiments conducted by Opportunity at Meridiani Planum. Science.

[28] Warner, N. H., et al. Geomorphology and origin of Homestead hollow, the landing location of the InSight lander on Mars. 50th Lunar and Planetary Science, Abstract #1184 (2019).

[29] Weitz, C. M. et al. Clast sizes and shapes at the InSight landing site. 50th Lunar and Planetary Science, Abstract #1392 (2019).

[30] Golombek M, Rapp D (1997). Size-frequency distributions of rocks on Mars and Earth analog sites: Implications for future landed missions. J. Geophys. Res..

[31] Golombek MP (2008). Size-frequency distributions of rocks on the northern plains of Mars with special reference to Phoenix landing surfaces. J. Geophys. Res. Planets.

[32] Golombek M (2012). Detection and characterization of rocks and rock size-frequency distributions at the final four Mars Science Laboratory landing sites. Mars.

[33] Golombek MP (2006). Geology of the Gusev cratered plains from the Spirit rover traverse. J. Geophys. Res. Planets.

[34] Grant, J. A. et al. Crater gradation in Gusev crater and Meridiani Planum, Mars. *J. Geophys. Res*. 10.1029/2005JE002465 (2006).

[35] Sweeney, J. et al. Degradation of 100-m-scale impact craters at the InSight landing site on Mars with implications for surface processes and erosion rates in the Hesperian and Amazonian. *J. Geophys. Res*. **123**, 2732–2759 (2018).

[36] Hartmann WK (1984). Does crater “saturation equilibrium” occur in the Solar System?. Icarus.

[37] Wilson, S. A. et al. Crater retention ages at the InSight landing site: Implications for the degradation history of Homestead hollow. 50th Lunar and Planetary Science, Abstract #2161 (2019).

[38] Warner, N. H. et al. Probing the regolith at the InSight landing site using rocky ejecta craters. 50th Lunar and Planetary Science, Abstract #1185 (2019).

[39] Grant JA (2004). Surficial deposits at Gusev crater along Spirit rover traverses. Science.

[40] Grant, J. A. et al. Modification of Homestead hollow at the InSight landing site based on the distribution and properties of local deposits. 9th Intl Conf. Mars, Abstract #6421 (2019).

[41] Banfield, D. et al. The atmosphere of Mars as observed by InSight. *Nat. Geosci.*10.1038/s41561-020-0534-0 (2020)

[42] Mehta M (2011). Explosive erosion during the Phoenix landing exposes subsurface water on Mars. Icarus.

[43] Williams, N. R. et al. Surface alteration from landing InSight on Mars and its implications for shallow regolith structure. 50^th^ Lunar and Planetary Science, Abstract #2781 (2019).

[44] Garvin, J. et al. Microtopography of the Mars InSight landing site: Geological implications. 50th Lunar and Planetary Science, Abstract #1705 (2019).

[45] Ansan, V. et al. InSight landing site: Stratigraphy of the regolith beneath the lander and in its surroundings, and implications for formation processes. 50th Lunar and Planetary Science, Abstract #1310 (2019).

[46] Sullivan R, Anderson R, Biesiadecki J, Bond T, Stewart H (2011). Cohesions, friction angles, and other physical properties of Martian regolith from Mars Exploration Rover wheel trenches and wheel scuffs, J. Geophys. Res..

[47] Delage P (2017). An investigation of the mechanical properties of some Martian regolith simulants with respect to the surface properties at the InSight mission landing site. Space Sci. Rev..

[48] Banin, A. et al. in *MARS* (eds Kieffer, H. H., Jakosky, B. M., Snyder,C. W. & Matthews, M. S.) 594–625 (University of Arizona Press, Tucson, 1992).

[49] Haskin LA (2005). Water alteration of rocks and soils from the Spirit rover site, Gusev crater, Mars. Nature.

[50] Hurowitz JA (2006). In situ and experimental evidence for acidic weathering of rocks and soils on Mars. J. Geophys. Res..

[51] Christensen, P. R., and H. J. Moore. in *MARS* (eds Kieffer, H. H., Jakosky, B. M., Snyder,C. W. & Matthews, M. S.) 686–727 (University of Arizona Press, Tucson, 1992).

[52] Herkenhoff, K. E. et al. in *The Martian Surface: Composition, Mineralogy and Physical Properties* (ed Bell III J. F.) 451–467 (Cambridge University Press, 2008).

[53] Golombek, M. P. et al. in *The Martian Surface: Composition, Mineralogy and Physical Properties* (ed Bell III J. F.) 468–497 (Cambridge University Press, 2008).

[54] Golombek, M. P. et al. The origin of sand on Mars. 49th Lunar and Planetary Science, Abstract #2319 (2018).

[55] Smith DE (2001). Mars Orbiter Laser Altimeter (MOLA): Experiment summary after the 1 year of global mapping of Mars. J. Geophys. Res..

[56] Yoder CF, Standish EM (1997). Martian precession and rotation from Viking lander range data. J. Geophys. Res..

[57] Le Maistre S (2016). InSight coordinates determination from direct-to-Earth radio-tracking and Mars topography model. Planet. Space Sci..

[58] Archinal BA (2018). Report of the IAU working group on cartographic coordinates and rotational elements 2015. Celest. Mech. Dyn. Astron..

[59] Golombek MP (2003). Rock size-frequency distributions on Mars and implications for MER landing safety and operations. J. Geophys. Res. Planets.

[60] Rosin P, Rammler E (1933). The laws governing the fineness of powdered coal. J. Inst. Fuel.

[61] Gilvarry JJ (1961). Fracture of brittle solids I. Distribution function for fragment size in single fracture (theoretical). J. Appl. Phys..

[62] Gilvarry JJ, Bergstrom BH (1961). Fracture of brittle solids II. Distribution function for fragment size in single fracture (experimental). J. Appl. Phys..

[63] Wohletz KH, Sheridan MF, Brown WK (1989). Particle size distributions and the sequential fragmentation/transport theory applied to volcanic ash. J. Geophys. Res..

[64] Brown WK, Wohletz KH (1995). Derivation of the Weibull distribution based on physical principles and its connection to the Rosin–Rammler and lognormal distributions. J. Appl. Phys..

[65] Charalambous, C. *On the Evolution of Particle Fragmentation with Applications to Planetary Surfaces*. PhD Thesis, Imperial College London (2014).

[66] Turcotte, D. L. *Fractals and Chaos in Geology and Geophysics*, 2nd edn. (Cambridge U. Press, Cambridge, 1997).

[67] Charalambous, C. et al. Rock distributions at the InSight landing site and implications based on fragmentation theory. 50th Lunar and Planetary Science, Abstract #2812 (2019).

[68] Heet TL, Arvidson RE, Cull SC, Mellon MT, Seelos KD (2009). Geomorphic and geologic settings of the Phoenix Lander mission landing site. J. Geophys. Res..

