# Supplementary material for: Geology of the InSight landing site on Mars
Source: Nat Commun. 2020 Feb 24;11:1014. doi: 10.1038/s41467-020-14679-1 (PMC7039939; doi:10.1038/s41467-020-14679-1)
Supplement: Supplementary file 1 — Supplementary Information [file 41467_2020_14679_MOESM1_ESM.pdf]

Supplementary Information

Geology of the InSight Landing Site on Mars

M. Golombek et al.,

Supplementary Table 1: Table of images acquired by InSight, Sols 0-247.

Total number of IDC images	1989
Total number of ICC images	801
Total number of all images	2790

Supplementary Table 2: Panorama/Mosaic Summary, Sols 0-247.

Panorama Type	Number of Images	Number of mosaics	Notes
Morning Panorama	38	1	Stereo coverage
Midday Panorama	219	4	Denser stereo coverage
Evening Panorama	62	2	Stereo coverage
Workspace from 1.5 meters away	113	2	~ 1.2 mm/pixel resolution, stereo
Workspace from 1 meter away	482	21	~ 0.8 mm/pixel resolution, stereo
Lander Above Deck	26	2	Pre- and post-instrument deployment
Lander Below Deck	12	4	Includes lander engine exhaust pits, in stereo
Total	947	34	

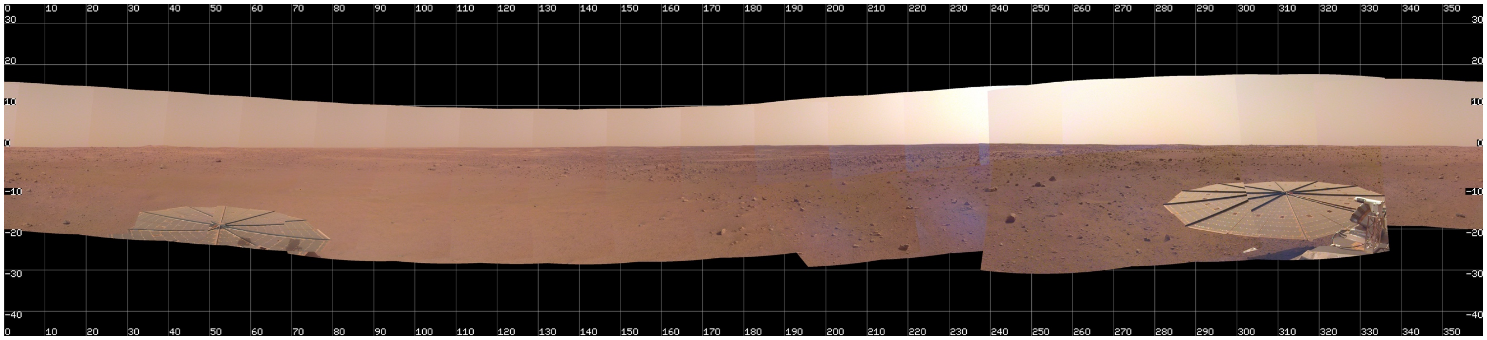

Supplementary Figure 1. Afternoon panorama of area around the InSight lander. Seam corrected afternoon panorama acquired between 15:00-16:45 Mars Local Mean Solar Time (LMST). This full 360° cylindrically projected mosaic is made up of 38 images (note azimuth and elevation grid). The frames making up this mosaic are color-calibrated products that have been adjusted for lighting conditions as if the Sun were at zenith on a clear day.

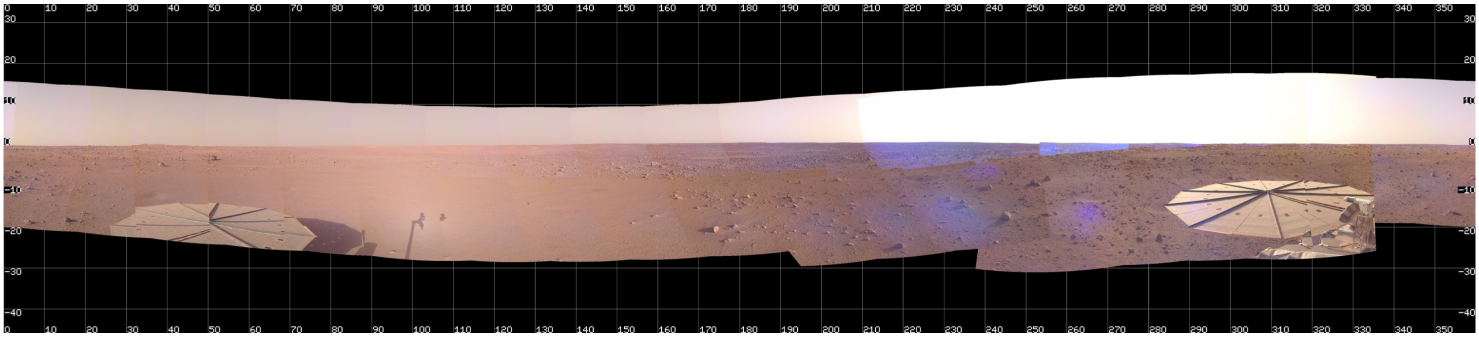

Supplementary Figure 2. Evening panorama of area around the InSight lander. Seam corrected evening panorama acquired between 16:45-17:45 Mars LMST. This full 360° cylindrically projected mosaic is made up of 38 images (note azimuth and elevation grid). The frames making up this mosaic are color-calibrated products that have been adjusted for lighting conditions as if the Sun were at zenith on a clear day.

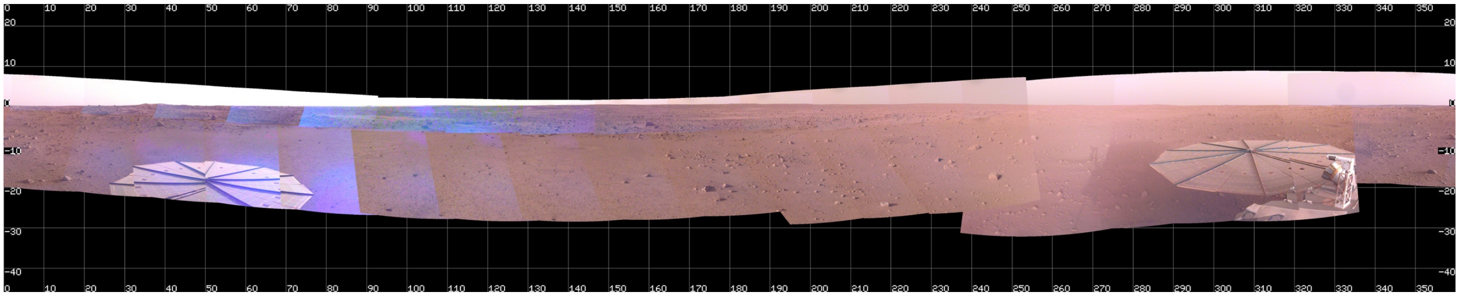

Supplementary Figure 3. Morning panorama of area around the InSight lander. Seam corrected morning Panorama acquired between 07:00-08:30 Mars LMST. This full 360 degree cylindrically projected mosaic is made up of 38 images (note azimuth and elevation grid). The frames making up this mosaic are color-calibrated products that have been adjusted for lighting conditions as if the Sun were at zenith on a clear day.

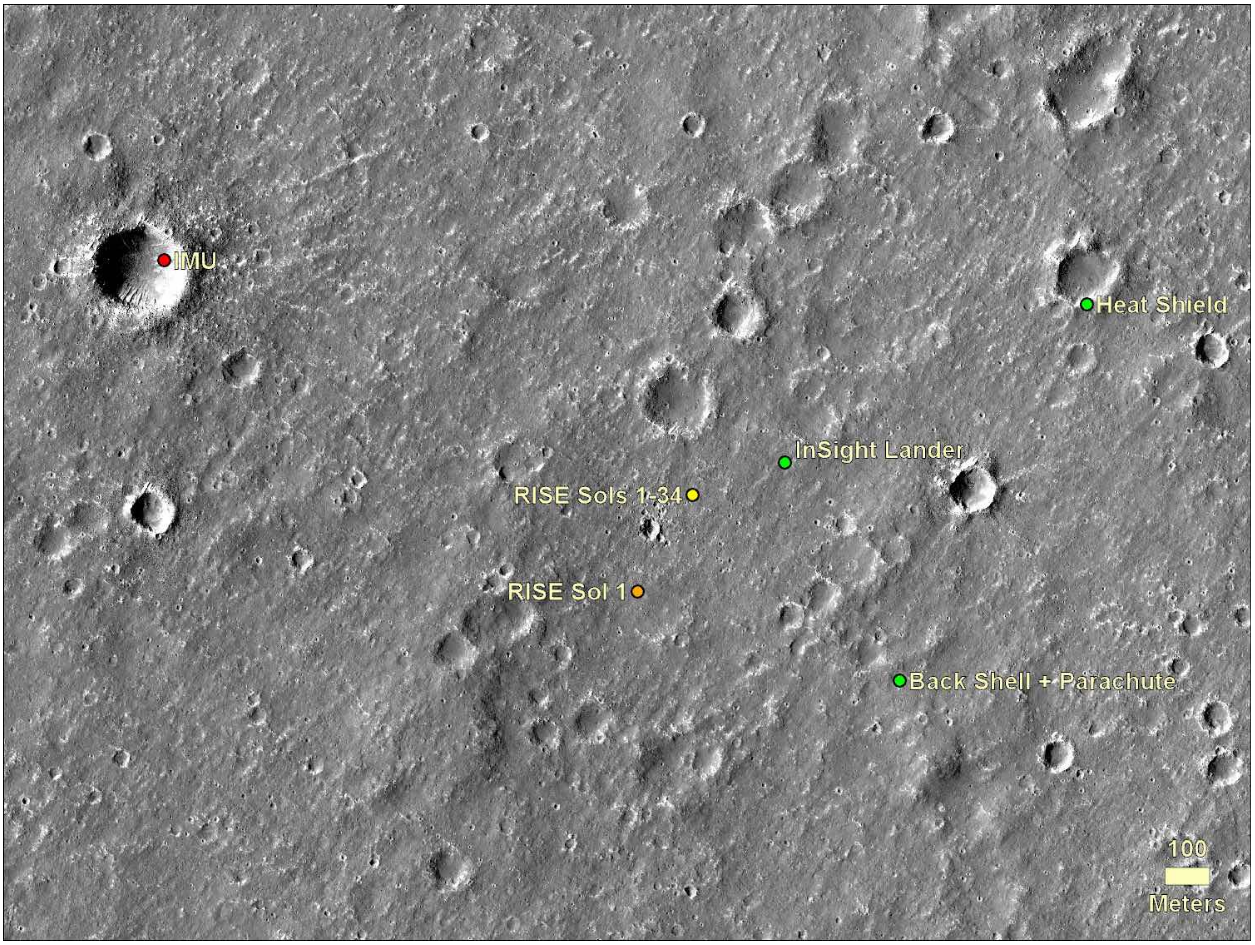

Supplementary Figure 4. Area around the lander with position estimates and hardware. Enlargement of area around the lander in HiRISE image showing the location of the lander, heatshield, and backshell (and parachute) and estimated positions from the IMU within the first few days after landing and RISE after the first day and after 34 sols of tracking with an improved rotation model (see Methods HiRISE and Doppler locations). HiRISE image ESP_036761_1845, which is one of the stereo pair used for the HiRISE digital elevation model of this area. North is up.

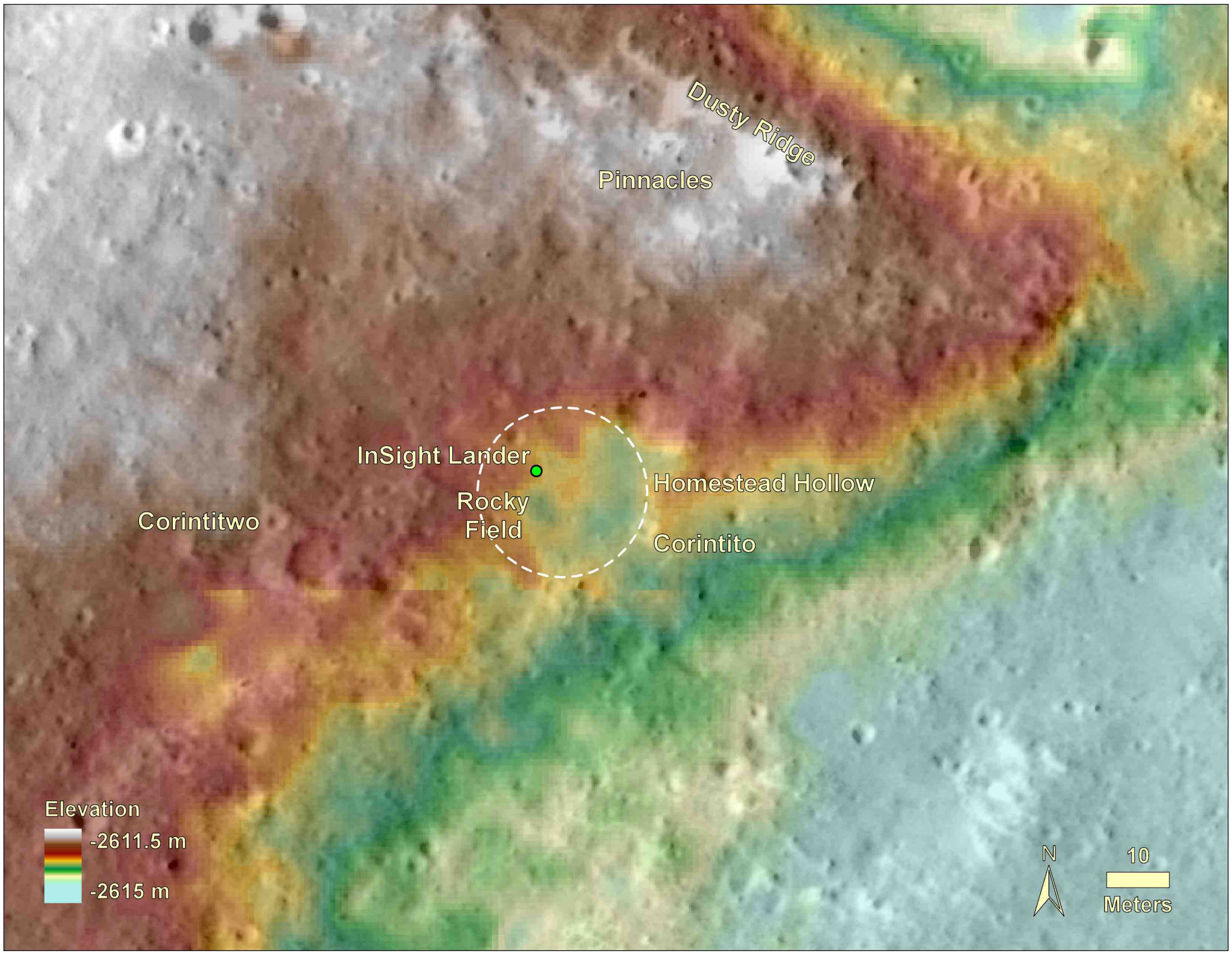

Supplementary Figure 5. Topographic map of Homestead hollow. Portion of HiRISE stereo derived digital elevation model (color) overlain on a HiRISE image of the area around the InSight lander (InSightE17_C)[1]. The lander is located in the northwest portion of a 27 m diameter quasi-circular depression, Homestead hollow (dashed circle). The hollow is around 0.3 m deep on average (varies from 0.2-0.5 m) with higher topography around it. Note slightly elevated, rougher and rockier Rocky field in the western portion of the hollow and the lower, smoother and less rocky smooth terrain in the rest of the hollow. Two Corinto secondary craters (Corintito and Corintitwo) are in view of the lander as are three rocks (The Pinnacles) and an eolian bedform (Dusty Ridge) to the northwest. Note craters in widely varying states of degradation from very fresh (Corinto secondaries) to highly degraded Homestead hollow.

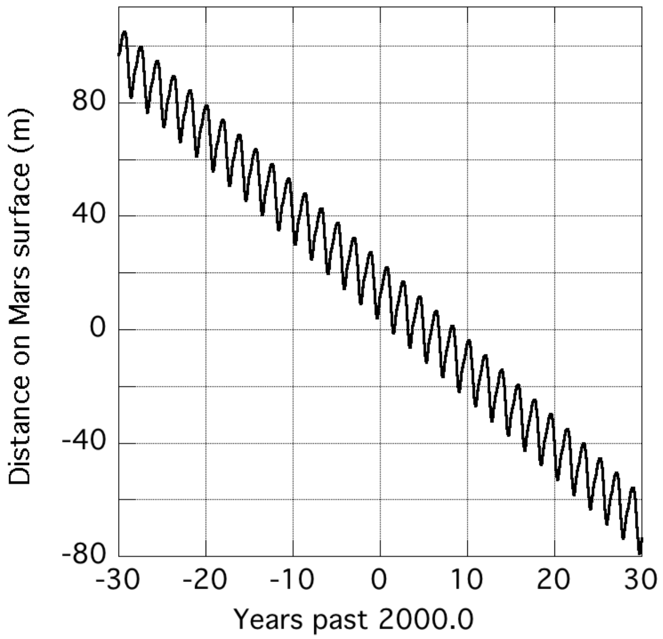

Supplementary Figure 6. Rotation model and Mars spin axis. Difference in inertial longitude or Mars prime meridian between Mars rotation model used for MOLA data reduction[2,3] and the Mars rotation model in Supplementary Table 3 for InSight.

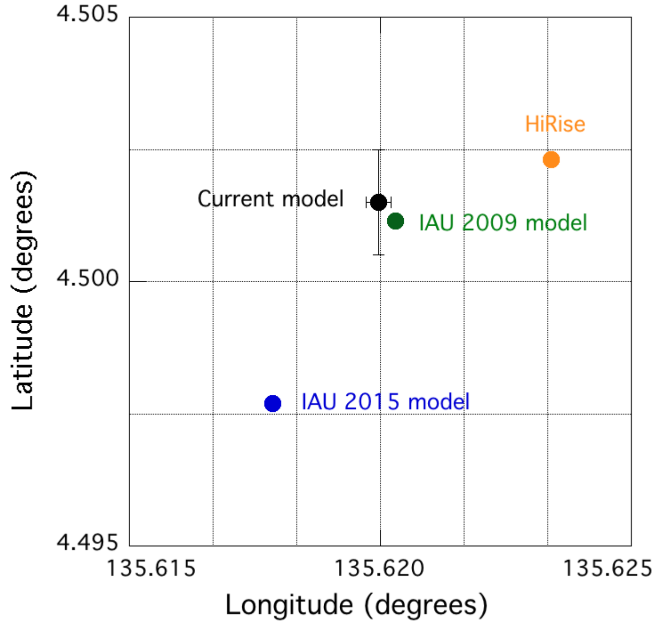

Supplementary Figure 7. Position estimates of InSight. Position of InSight derived from the first 34 sols of Doppler data using three Mars rotation models, and as derived from images of the lander in HiRISE in the cartographic frame. The uncertainty in longitude is due to the Doppler data noise and the uncertainty in latitude is due to the variation in surface topography over the longitude uncertainty range (see Methods HiRISE and Doppler locations).

Supplementary Table 3. Expressions for Mars Rotation Angles.

α_0	=	317.681115 - 0.10865304T
	+	0.000068*sin(199.0 + 19139.4819985T)
	+	0.000238*sin(226.3 + 38280.8511281T)
	+	0.000052*sin(249.7 + 57420.7251593T)
	+	0.000009*sin(266.2 + 76560.6367950T)
δ_0	=	52.886352 - 0.06157491T
	+	0.000051*sin(122.4 + 19139.9407476T)
	+	0.000141*sin(43.1 + 38280.8753272T)
	+	0.000052*sin(249.7 + 57420.7251593T)
	+	0.000009*sin(266.2 + 76560.6367950T)
W	=	176.630 + 350.891982442944d
	+	0.000138*sin(125.4 + 19140.0328244T)
	+	0.000160*sin(37.4 + 38281.0473591T)
	+	0.000040*sin(56.7 + 57420.9295360T)
	+	0.000005*sin(60.0 + 76560.2552215T)
	+	0.000001*sin(104.9 + 95700.4387578T)

Rotation angles in degrees; α_0 is the right ascension and δ_0 the declination of the spin axis and W the rotation about the spin axis[4], where T is time in Julian centuries (36,525 days) and d is time in days from the epoch 2000 January 1 12 h TDB (barycentric dynamical time).

Supplementary Table 4. InSight Doppler Positions.

sol 1	R_s	=	3382.6136	± 0.001 km
	λ	=	135.61996	± 0.00025 deg
	R_z	=	266.286	± 0.062 km
	ϕ	=	4.5015	± 0.001 deg
sol 34	R_s	=	3382.6137	± 0.0002 km
	λ	=	135.61994	± 0.00025 deg
	R_z	=	266.286	± 0.050 km
	ϕ	=	4.5011	± 0.0008 deg

Estimated InSight position coordinates from Doppler tracking data from the first Martian day after landing, sol 1, and from the first 34 sols of data. R_s is the distance from the spin axis; λ is the longitude; R_z is the distance from the equatorial plane; ϕ is the planetocentric (spherical) latitude.

Supplementary Note 1

Perched versus Partially Buried Rocks

A sense of where exhumation or burial has occurred can be gained by characterizing the exposed cross-section of rocks as a proxy for visually determining whether they are mostly exposed, partially exposed, or mostly buried. This approach assumes the shape of fragments larger than ~5 cm across at Homestead hollow is broadly similar to the mostly equant shape of rocks at Lonar crater that formed into basalt[5] (same as the landing site). A mosaic covering approximately 180 degrees from the north to east to south side of lander in Homestead hollow (from left to right) is shown in Supplementary Figure 8. Colored dots denote the relative distribution of buried (only top exposed with minimal relief, red), embedded (multiple faces exposed with moderate relief, yellow), and perched (nearly complete exposure with portion of base visible, green) rocks in and around the hollow. 1180 rocks were examined within and around the half circumference of the hollow (333 on rim, 847 in the interior). Large rocks are easier to detect on the rim, whereas smaller rocks are easier to detect in the hollow and closer to the lander (and contribute to the greater number of rocks detected within the hollow). Perched rocks represent ~70% of those seen on the rim relative to a combined 30% buried (1%) and embedded (29%) rocks. By contrast, the combined 58% of buried (9%) and embedded (49%) rocks is greater than the 42% perched rocks mapped inside the hollow. Note that rocks in foreground can block those in background, and the view favors detection of small rocks within hollow and hampers detection of perched and buried rocks at a distance (where the base is often obscured by other rocks). Moreover, the viewing angle may preclude detection of buried rocks near and beyond edge of the hollow and probably explains the apparent paucity of buried rocks beyond the near field and especially along the rim. Nevertheless, the large increase in perched rocks relative to embedded and buried rocks along and beyond the hollow rim and the comparable dominance of buried and embedded rocks relative to perched rocks inside the hollow appears real (Supplementary Figure 8).

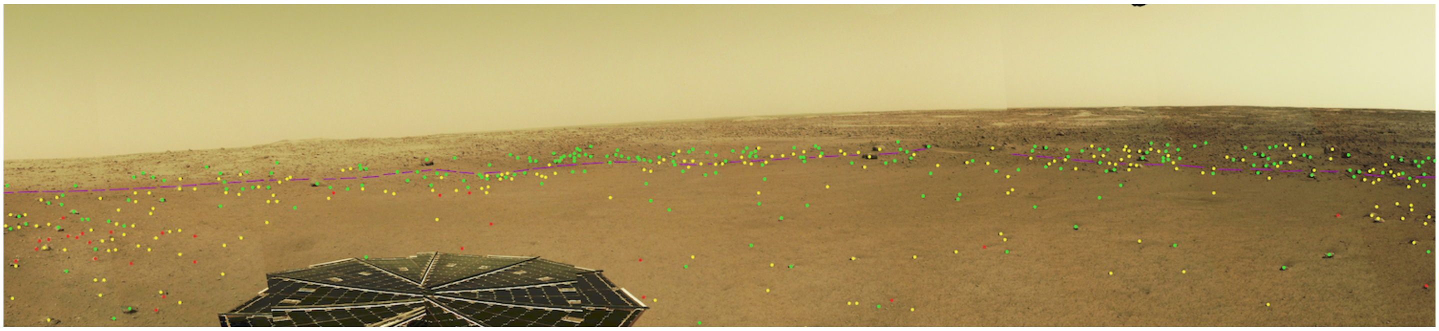

Supplementary Figure 8. Perched versus buried rocks. Mosaic covering 180° showing buried, embedded and perched rocks. Colored dots denote the relative distribution of buried (only top exposed with minimal relief, red), embedded (multiple faces exposed with good relief, yellow), and perched (nearly complete exposure with portion of base visible, green) rocks in and around the hollow. The purple line is the edge of the hollow. Portion of IDC Mosaic D_LRGB_0014_RAS030100CYL_R__SCIPANQM1.

Supplementary Note 2

Terrain Strength from Elastic Response to Wind Vortices

Convective vortices (named dust devils when the vortex transports dust particles) are detected as a sharp dip in local pressure in the time series. The decrease in pressure by the convective vortex pulls the elastic ground up during its passage causing the surface to tilt away from the vortex. This leads to a tilt signature on the horizontal component of a seismometer in contact with the ground. The isolated seismic signature of a convective vortex was first detected on Earth in 2015[6] and have since been detected on Mars with SEIS[7,8]. The dust devil tracks observed around the InSight landing site in HiRISE images[9] indicate a preferential direction, aligned closely with the most common wind direction (~N145°E ±30°). From atmospheric observations and modeling in sites similar to the InSight landing site (without significant topographic or albedo contrasts), an equal number of vortices are expected to the east compared to those to the west. This has been confirmed by analyses of HiRISE satellite images showing that the dust devil tracks pass nearby on both sides of InSight giving preferential tilt directions at the vortex closest approaches of ~N55E° and ~N125W° (+/- 30°) (Supplementary Figure 9).

By studying the direction of the ground tilt (measured by SEIS[10]) at the closest approach (when radial tilt amplitude is largest) it appears that the majority of seismically detected vortices have a closest approach to the east of SEIS and that there is a distinct lack of detections to the west (Supplementary Figure 9).

Wind drag[11] from the vortex winds was considered as a possible source of this azimuth bias. On the horizontal axes, the effect of the wind drag will likely not be the same for a dust devil passing on one side or the other, if there is an a priori dominant rotation direction of the dust-devil convective vortices. There is, however, no such dominant rotation as was hinted at from the early field observations of dust devils in terrestrial deserts[12] and Viking Lander observations on Mars[13,14]. In other words, vortex rotation is equally divided between clockwise and counter-clockwise. This is consistent with vorticity being generated at very local scales where the Coriolis force is negligible even for the largest dust-devil-like convective vortices. In addition, at closest approach, the tangential wind velocity is perpendicular to the direction of the tilt and any drag force would not influence the seismic measurement in the tilt direction. Given the position of SEIS to the south of the InSight lander, it is also possible that the lander itself may perturb the vortex trajectories. However, if this effect exists, it should only be important for direct vortex encounters (i.e., when the center of the vortex passes directly over the lander).

Lower strength ground will deform more as a vortex passes, leading to a larger ground deformation[15]. This larger ground deformation will be detected more easily by SEIS. Therefore, our preferred explanation for the observed close-approach azimuth bias is that the ground has lower strength to the east thus increasing the number of seismic detections of vortices on that side. Such an interpretation of lower strength ground to the east is consistent with the infill of Homestead hollow with predominantly sand by eolian activity and the position of the lander (and SEIS) on the western edge of the hollow.

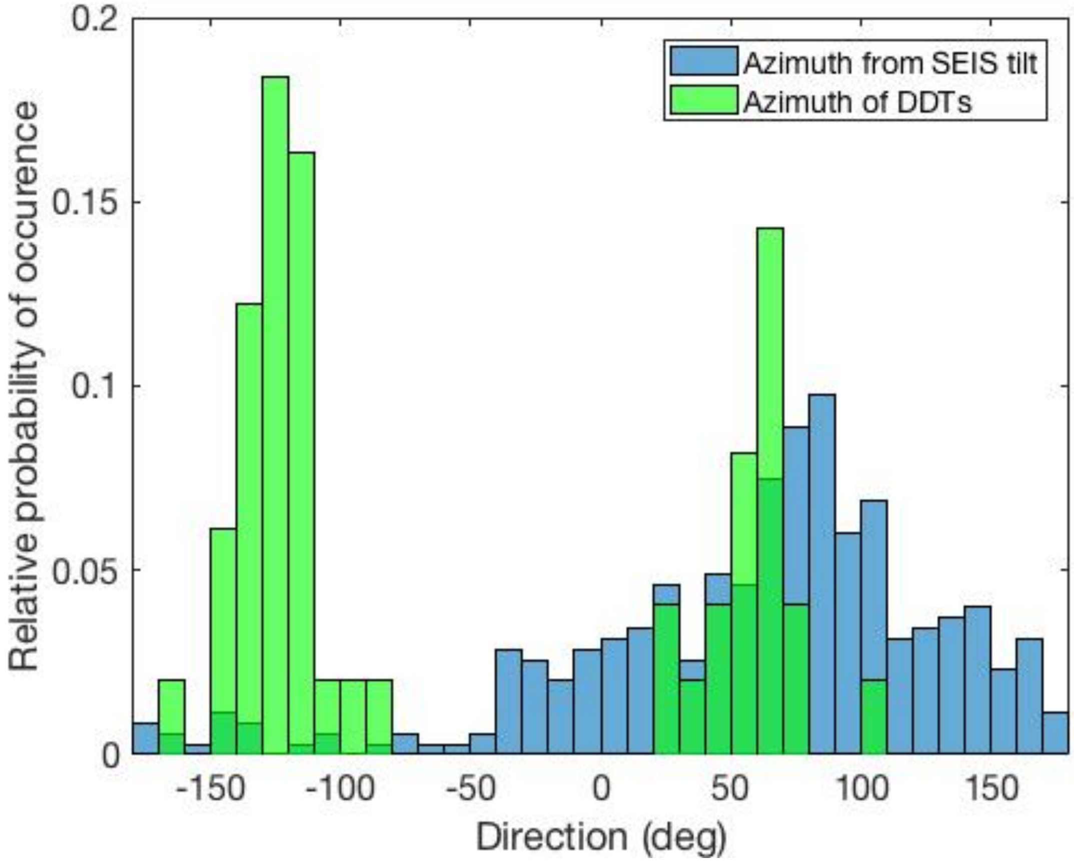

Supplementary Figure 9. Azimuths of wind vortices. Comparison of the distribution of wind vortices azimuths at the point of closest approach as measured from the SEIS tilt (blue), and measured from dust devil tracks (DDTs) nearby the lander in HiRISE images (green). The SEIS tilt results are shown here for 349 pressure drop events between sols 73 and 180. The events were selected based on simultaneous detections of the vortex passage in both the pressure and seismic data, ensuring that the pressure drop was typical of a convective vortex (high quality of a Gaussian fit to the pressure profile). The DDT results shown here are for track detections made in the close vicinity of the lander (49 tracks in an area of about 12 km^2 around the lander). The relative probability of occurrence is the number of elements in the histogram bin divided by the total number of elements in the input data. The "dark part" of the bars shows the blue SEIS tilt data underneath the green DDT data.

Supplementary Note 3

Soil Cohesion Estimates from Slope Stability Analysis

Cohesion estimates are obtained using slope stability analysis for the pits observed beneath the InSight lander thrusters and near the HP[3] mole. We assume plane strain conditions, that the material is homogeneous, and that a Mohr-Coulomb failure criteria is satisfied along the failure surfaces. Two failure mechanisms are examined: a straight-line failure plane (Supplementary Figure 10a) using the Culmann method[16] and a circular arc failure plane (Supplementary Figure 10b) using stability factor charts[16,17]; both failure planes pass through the toe of the slope.

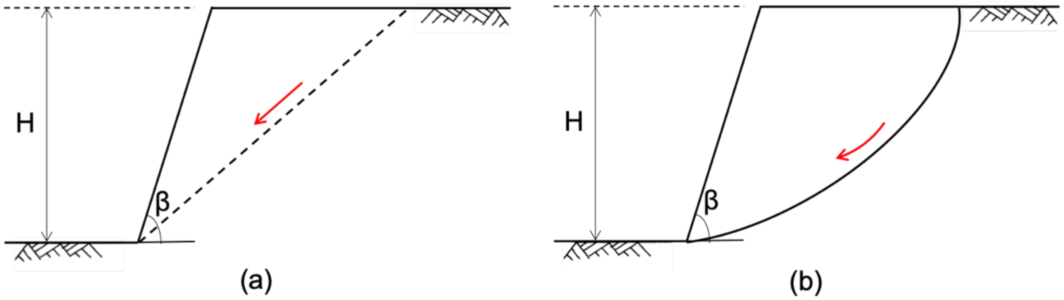

Supplementary Figure 10. Slope Failure Surfaces. Slope failure surfaces used in stability calculations: (a) straight line and (b) circular arc. H is the height of the slope, β is the slope inclination angle, and the red arrows correspond to the direction of the movement.

The Culmann method considers that static equilibrium conditions are satisfied and provides a relationship between the resisting force (i.e., the shear strength of the soil along the failure surface) and the driving force (i.e., the weight W of the region isolated by the failure surface). As a result, the stability of the entire slope can be simply represented by:

$$\frac{\rho g H}{c} = \frac{4 \sin\beta \cos\phi}{1 - \cos(\beta - \phi)} \tag{1}$$

where ρ is the bulk density of the soil, g is the gravitational acceleration, and H is the height of the slope, c is the cohesion, ϕ is the angle of internal friction, and β is the slope inclination angle. The term $\rho g H/c$ is a dimensionless expression called the stability factor and describes an equilibrium criterion for which the slope is marginally stable. For a circular-arc failure plane, we use the chart derived in Moore et al.[17] [Figure 66] that plots the stability factor as a function of the slope angle for different values of internal friction angle.

Slope inclination angle β and height H of the pits underneath the lander are taken from elevation profiles in stereogrammetric digital elevation models[18] (Supplementary Figure 11). Slope stability analysis is conducted on two profiles, which have the largest slope inclination angle (β = 66°, H = 0.048 m; G in Supplementary Figure 6) and the largest slope height (β = 57°, H = 0.073 m; H in Supplementary Figure 11). Cohesion estimates are deduced from the stability factor assuming a range of internal friction angles ϕ between 30° and 50° and bulk density ρ between 1200 kg/m^3 and 1600 kg/m^3. We emphasize that the computed cohesion values are minimum estimates. The

results show that a circular-arc failure plane yields higher minimum values of cohesion than a straight-line failure plane, which are typically used for steep slopes. For a circular-arc failure plane, the cohesion must exceed 5 to 24 Pa in order to maintain the slopes observed in the pits underneath the lander thrusters.

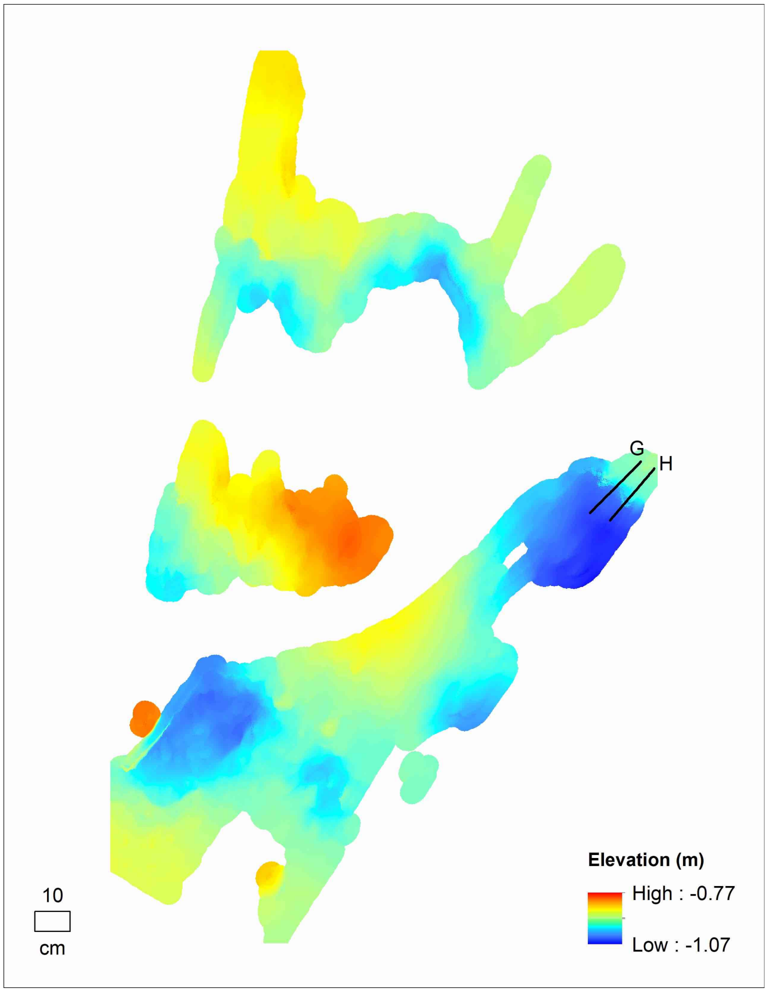

Supplementary Figure 11. Topography beneath lander. Digital elevation model of part of the area underneath the lander. Gaps in the map are where stereo correlations are poor due to highly oblique viewing geometry and lander hardware obscurations. Where stereo correlations were calculated but sparse, elevations were interpolated from neighboring points within 3 cm. Profiles were taken in the direction of steepest topographic gradient with average slopes calculated for the steepest sections of each profile; the steepest measured were profiles G and H at 66° and 57°, respectively. The map has elevation postings every 1.2 mm and was created from images D001L0018_598131526EDR_F0606_0010M2 and D001R0018_598131636EDR_F0606_0010M2.

Cohesion estimates are also obtained for the pit that formed around the HP^3 mole. On Sol 240, the flat part of the IDA scoop was used to apply a preload at the edge of the HP^3 pit in an attempt to

cause failure of the western wall, as shown in Supplementary Figure 12a. The IDA flight software[19] determined that the force applied by the scoop was F_z = 59 N in the vertical direction and F_r = 40 N in the radial direction, which did not cause slope failure. The force F_r, which acts away from the lander, does not affect the stability and only the vertical force F_z is considered in the analysis. The force P acting on the region isolated by the failure surface due to the scoop preload is given by:

$$P = pd = \frac{H\,F_z}{w_s\,l_s}\left[\cot\left(\frac{\beta+\phi}{2}\right) - \cot\beta\right] \tag{2}$$

where, as depicted in Supplementary Figure 12b, p is the uniform stress exerted by the scoop given by $p = F_z/w_s\,l_s$ and d is the length of the region isolated by the failure surface on which the scoop preload is applied given by $d = H\left[\cot\left(\frac{\beta+\phi}{2}\right) - \cot\beta\right]$, w_s = 0.071 m is the width of the scoop, and l_s = 0.092 m is the length of the scoop. Considering that the force P adds to the downward force, a closed-form expression for the cohesion c derived using the Culmann method is:

$$c = \frac{(W+P)(\cos\phi - \cos\beta)}{2\mathrm{H}\cos\,\phi} \tag{3}$$

where the weight W of the region isolated by the failure surface is given by:

$$W = \frac{1}{2}\rho g H^2 \frac{\sin\left(\frac{\beta-\phi}{2}\right)}{\sin\left(\frac{\beta+\phi}{2}\right)\sin\beta} \tag{4}$$

The HP^3 pit is assumed to have a slope inclination angle β = 80° and a height H = 0.05 m. Minimum estimates of the cohesion obtained using equation (3) are calculated for a range of internal friction angles ϕ between 30° and 50° and bulk densities ρ between 1200 kg/m^3 and 1600 kg/m^3. The results imply that a minimum cohesion c between 1 and 1.9 kPa is required for the slope to be marginally stable when a force P is applied by the IDA scoop. Slope stability analysis for the HP^3 pit performed without the IDA scoop preload or considering the load due to the right front foot of the HP^3 instrument, immediately adjacent to the pit, yields lower cohesion estimates.

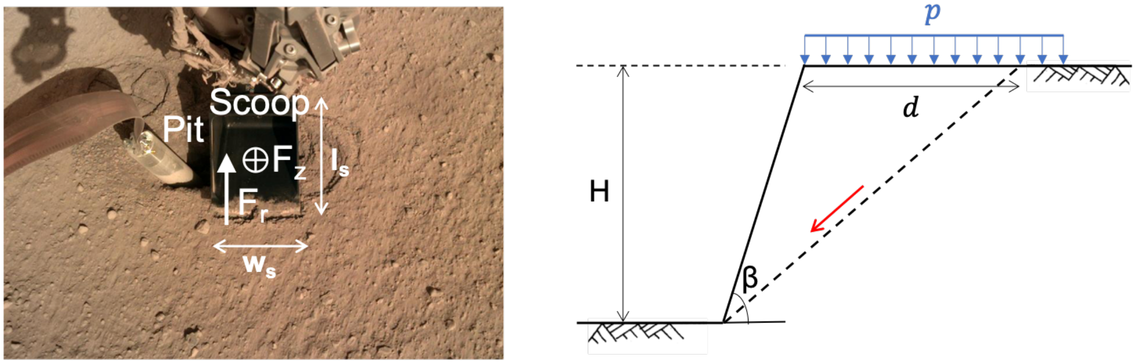

(a) (b)

Supplementary Figure 12: Scoop Surface Interactions. (a) Annotated image of the scoop interacting with the soil near the HP[3] pit. Direction of the vertical force F_z and radial force F_r during the preload. The dimensions of the scoop width and length are, w_s and l_s, respectively. (b) Straight-line failure plane with uniform pressure p (blue arrows). H is the height of the slope, β is the slope inclination angle, d is the length of the region isolated by the failure surface on which the scoop preload is applied, and the red arrow corresponds to the direction of the movement.

Supplementary Note 4

RAD Measurements and Thermal Inertia

We use brightness surface temperatures acquired by the HP[3] radiometer at 8-14 μm from under the lander deck to derive the thermal inertia of the soil in the far RAD spot, which is least influenced by the lander[20]. First, we average the measurements in hourly bins (1 hour is 1/24th of a sol) to minimize spread and processing time. Then, we fit the temperatures acquired at local times most diagnostic of thermal inertia (e.g., 12AM-Dawn and 11AM-2PM) with a numerical model's output[21] where the surface albedo and regolith thermal inertia are free parameters. Key inputs include the atmospheric dust opacity as derived from images of the sky[8], the local slope, slope azimuth, latitude, elevation etc. An example of diurnal temperature fit is presented in Supplementary Figure 13.

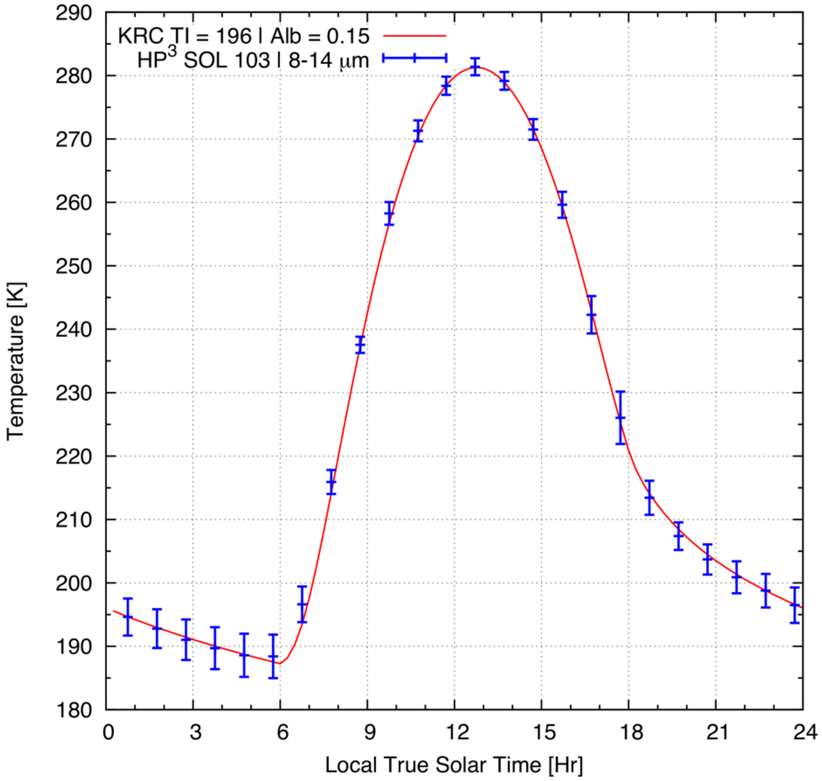

Supplementary Figure 13: Thermal inertia from diurnal temperature. Example of diurnal temperature fit, sol 103, albedo = 0.15, thermal inertia = 196 J m^{-2} K^{-1} $s^{-1/2}$. Error bars correspond to reported instrumental uncertainty. Data acquired near the AM and PM crossover times, i.e., when the temperature is rapidly changing are not used for the fit.

A similar model based on the single column version of the LMD General Circulation Model full physics package as described in Forget et al.[22] and Pottier et al.[23] arrives at similar values of thermal inertia and albedo. Best fits are obtained with a homogeneous regolith characterized by typical thermal inertia values in the range of 160-230 J m^{-2} K^{-1} $s^{-1/2}$ and albedo of 0.13-0.15.

Inertia derivations are generally accurate within ±15%[24,25], although in the present case, the excellent knowledge of the surface properties and multiple observations at various local times certainly indicate an accuracy closer to 10%. Based on in situ observations[25,26,27] and laboratory measurements[28], such values are associated with unconsolidated fine to medium sand size material, with typical grain sizes of ~130-350 μm. The volumetric amount of cementing phase can hardly be constrained for such low inertia values, but it must be <1% in volume (or more if not located in the inter-grain regions, i.e., not participating in the thermomechanical properties of the regolith). For comparison, ~1% cement in volume raises thermal inertia values by large factors (i.e., ~600 J m^{-2} K^{-1} $s^{-1/2}$)[29,30]. No indication of layering is discernable from the diurnal temperature trends, although very fine (sub mm) or deep layering (sub dm) may be unveiled from future analysis of eclipse or seasonal data trends.

Supplementary References:

1. Fergason, R., Kirk, R. L., et al. Analysis of local slopes at the InSight landing site on Mars, Space Science Reviews, 211, 109-133 (2017).

2. Smith, D., G. Neumann, R. E. Arvidson, E. A. Guinness, and S. Slavney, Mars Global Surveyor Laser Altimeter Mission Experiment Gridded Data Record, NASA Planetary Data System, MGS-M-MOLA-5-MEGDR-L3-V1.0 (2003).

3. Archinal, B. A., M. F. A'Hearn, E. Bowell, A. Conrad et al. Report of the IAU working group on cartographic coordinates and rotational elements. Celestial Mechanics and Dynamical Astronomy, 109(2):101 (2011).

4. Konopliv, A. S., R. S. Park, W. M. Folkner. An improved JPL Mars gravity field and orientation from Mars orbiter and lander tracking data. Icarus 274, 253-260 (2016)

5. Kumar, P. S., Prasanna Lakshmi, K. J., Krishna, N., Menon, R., Sruthi, U., Keerthi, V., Senthil Kumar, A., Mysaiah, D., et al. Impact fragmentation of Lonar crater, India: Implications for impact cratering processes in basalt. J. Geophys. Res., 119, 2029–2059 (2014).

6. Lorenz, R. D., et al. Seismometer detection of dust devil vortices by ground tilt. Bulletin of the Seismological Society of America 105, 5, 3015-3023, 10.1785/0120150133 (2015).

7. Lognonné, P. et al. Initial results from SEIS with a focus on shallow Mars structure. Nature Geosciences, published simultaneously, doi.org/10.1038/s41561-020-0536-y (2020).

8. Banfield, D., A. Spiga et al. The atmosphere of Mars as observed by InSight. Nature Geosciences, published simultaneously, DOI: 10.1038/s41561-020-0534-0 (2020)

9. Golombek, M., et al. Geology and physical properties investigations by the InSight Lander. Space Science Reviews 214: 84 (2018).

10. Lognonné, P., W. B. Banerdt, D. Giardini et al., SEIS: Insight's Seismic Experiment for Internal Structure of Mars, Space Science Reviews 215: 12 (2019).

11. Murdoch et al., Evaluating the wind-induced mechanical noise on the InSight seismometers. Space Science Reviews 211, 1-4, 429-455 (2017)

12. Balme, M., and R. Greeley. Dust devils on Earth and Mars. Reviews of Geophysics, 44, 3, RG3003 (2016)

13. Murphy et al., Field Measurements of terrestrial and martian dust devils. Space Science Reviews 203, 1-4, 39-87 (2016)

14. Ryan, J. A. and R. D. Lucich, Possible dust devils, vortices on Mars. J. Geophys. Res. 88, 11005-11011 (1983)

15. Kenda, B. et al. Modelling of ground deformation and shallow surface waves generated by martian dust devils and perspectives for near-surface structure inversion. Space Science Reviews 211, 1-4, 501-524 (2017)

16. Scott, R.F. Principles of soil mechanics, Addison-Wesley Publishing Co. (1963).

17. Moore, H.J., Hutton, G.D., and Spitzer, C.R. Physical properties of the Surface materials at the Viking landing sites on Mars. U.S. Geol. Surv. Pap. 1389: 222pp, (1987).

18. Abarca, H., R. Deen et al. Image data processing for the InSight lander operations and science. Space Science Reviews 215: 22 (2019).

19. Trebi-Ollennu, A., W. Kim, K. Ali et al. InSight Mars lander robotics instrument deployment system. Space Science Reviews 214: 93 (2018).

20. Spohn, T. et al. The heat flow and physical properties package (HP^3) for the InSight mission. Space Science Reviews 214: 96 (2018).

21. Kieffer, H. Thermal model for analysis of Mars infrared mapping, Journal of Geophysical Research: Planets 118(3), 451-470 (2013).

22. Forget, F., Hourdin, F., Fournier, R., Hourdin, C., Talagrand, O., Collins, M., Lewis, S. R., Read, P. L., and Huot, J.-P. Improved general circulation models of the Martian atmosphere from the surface to above 80 km. J. Geophys. Res. 104 (E10), 24155–24175 (1999).

23. Pottier, A., Forget, F., Montmessin, F., Navarro, T., Spiga, A., Millour, E., Szantai, A., Madeleine, J.-B. Unraveling the martian water cycle with high-resolution global climate simulations. Icarus 291, 82-106 (2017).

24. Fergason, P. R. Christensen, and H. H. Kieffer. High resolution thermal inertia derived from THEMIS: Thermal model and applications. Journal of Geophysical Research 111, E12004 (2006).

25. Fergason, P.R.Christensen, J. F. Bell III, M. P. Golombek, K. E. Herkenhoff, and H. H. Kieffer. Physical properties of the Mars Exploration Rover landing sites as inferred from Mini-TES-derived thermal inertia. Journal of Geophysical Research 111, E02S21 (2006).

26. Edwards, C. S., S. Piqueux, V. E. Hamilton, R. L. Fergason, K. E. Herkenhoff, A. R. Vasavada, K. A. Bennett, L. Sacks, K. Lewis, and M. D. Smith. The thermophysical properties of the Bagnold Dunes, Mars: Ground-truthing orbital data. Journal of Geophysical Research-Planets 123(5), 1307-1326 (2018).

27. Vasavada, A. R., S. Piqueux, K. W. Lewis, M. T. Lemmon, and M. D. Smith. Thermophysical properties along Curiosity's traverse in Gale crater, Mars, derived from the REMS ground temperature sensor. Icarus 284, 372-386 (2017).

28. Presley, M. A., and P. R. Christensen. Thermal conductivity measurements of particulate materials, Part II: Results. Journal of Geophysical Research, 102, 6551-6566 (1997).

29. Mellon, M. T., R. L. Fergason, and N. E. Putzig. The thermal inertia of the surface of Mars, in The Martian Surface: Composition, Mineralogy, and Physical Properties, edited by J. F. Bell III, Cambridge University Press (2008).

30. Piqueux, S., and P. R. Christensen. A model of thermal conductivity for planetary soils: 2. Theory for cemented soils. Journal of Geophysical Research 114, doi:E09006 10.1029/2008je003309 (2009).
